# Quantitative Analysis of Retinal Structure and Function in Two Chromosomally Altered Mouse Models of Down Syndrome

**DOI:** 10.1167/iovs.61.5.25

**Published:** 2020-05-16

**Authors:** Daniella B. Victorino, Jonah J. Scott-McKean, Mark W. Johnson, Alberto C. S. Costa

**Affiliations:** ^1^ Division of Pediatric Neurology, Department of Pediatrics, Case Western Reserve University, Cleveland, Ohio, United States; ^2^ Postgraduate Program in Neurology and Neuroscience, Federal University of São Paulo, São Paulo, Brazil; ^3^ Department of Psychiatry, Case Western Reserve University, Cleveland, Ohio, United States

**Keywords:** Down syndrome, trisomy 21, Ts65Dn mice, Dp(16)1Yey/+ mice, Dp16 mice, retina, optical coherence tomography (OCT), ketamine, tribromoethanol, urethane, fluorescein angiography, electroretinogram (ERG)

## Abstract

**Purpose:**

Ophthalmic disorders are among the most prevalent Down syndrome (DS) comorbidities. Therefore, when studying mouse models of DS, ignoring how vision is affected can lead to misinterpretation of results from assessments dependent on the integrity of the visual system. Here, we used imaging and electroretinography (ERG) to study eye structure and function in two important mouse models of DS: Ts65Dn and Dp(16)1Yey/+.

**Methods:**

Cornea and anterior segment were examined with a slit-lamp. Thickness of retinal layers was quantified by optical coherence tomography (OCT). Eye and lens dimensions were measured by magnetic resonance imaging (MRI). Retinal vasculature parameters were assessed by bright field and fluorescent imaging, and by retinal flat-mount preparations. Ganzfeld ERG responses to flash stimuli were used to assess retinal function in adult mice.

**Results:**

Total retinal thickness is significantly increased in Ts65Dn and Dp(16)1Yey/+ compared with control mice, because of increased thickness of inner retinal layers, including the inner nuclear layer (INL). Increased retinal vessel caliber was found in both chromosomally altered mice when compared with controls. ERG responses in Ts65Dn and Dp(16)1Yey/+ mice showed subtle alterations compared with controls. These, however, seemed to be unrelated to the thickness of the INL, but instead dependent on the anesthetic agent used (ketamine, tribromoethanol, or urethane).

**Conclusions:**

We provide evidence of retinal alterations in Ts65Dn and Dp(16)1Yey/+ mice that are similar to those reported in persons with DS. Our ERG results are also a reminder that consideration should be given to the choice of anesthetic agents in such experiments.

Down syndrome (DS) is the most frequent cause of intellectual disability of genetic origin.^1^ This disorder is generally caused by the trisomy of chromosome 21^2^ and affects approximately one in every 700 live births in the United States.^3^ Individuals with DS typically display a wide range of phenotypes and comorbidities, which include a variety of ophthalmic disorders.

Abnormalities in eye structure and function, including refractive errors, cataracts, accommodative insufficiency, and strabismus are considered to be the main causes of the visual deficits observed in this population.^4^^–^^11^ However, central nervous system abnormalities of visual pathways have also been invoked to explain deficits in visual function in individuals with DS.^12^^–^^16^ At the level of the retina, for example, there have been reports of increased retinal thickness^17^ and greater number of retinal blood vessels radiating from the optic disc^18^^–^^23^ in persons with DS compared with individuals without DS. Intermediate-level visual processing abnormalities have also been reported, including abnormal visual evoked potentials (VEPs) in children^13^^,^^14^^,^^24^^,^^25^ and adults with DS.^14^^,^^24^^,^^26^

Mouse models of DS have been essential tools for investigating the molecular pathogenesis and pathophysiology of DS. In particular, the segmentally trisomic Ts65Dn mouse has been the most widely used mouse model of DS and is the most complete in terms of mimicking phenotypes seen in persons with DS, including impaired learning and memory.^27^ Ts65Dn mice are trisomic for most of the orthologous human chromosome 21 genes conserved in the distal segment of mouse chromosome 16.^28^ Arguably, however, the main shortcomings of these mice as a model of DS are the incompleteness and partial nonspecificity of the set of human chromosome 21 orthologous genes contained in the Ts65Dn trisomic segment. For instance, Ts65Dn mice are also trisomic for a >5.8Mb subcentromeric region on mouse chromosome 17 (containing at least 50 genes) that is not orthologous to any region on human chromosome 21.^29^

Another commonly studied mouse model of DS is the Dp(16)1Yey+ (Dp16), which was chromosomally engineered to carry a duplication of the complete human chromosome 21-orthologous region of mouse chromosome 16.^30^^,^^31^ Although Dp16 mice do not parallel all the DS-like phenotypes observed in Ts65Dn mice, they display many DS-like traits, such as deficits in motor function^32^ and learning and memory deficits.^31^^,^^33^

Given the prevalence of ophthalmic disorders in DS, it is somewhat surprising how few studies have been published on the visual system of mouse models for this genetic disorder. One potential consequence of this dearth of understanding of how vision is affected in these mouse models would be the misinterpretation of behavioral data obtained through testing protocols that are significantly dependent on the integrity of the visual system.

Several years ago, work from our research group showed that Ts65Dn mice exhibit deficits in luminance threshold, spatial resolution, and contrast threshold compared with euploid control mice, as assessed by pattern visual-evoked potentials (VEPs) recorded from the primary visual cortex.^34^ We also found that basic retinal function was preserved in these mice, given that the analysis of the B-wave of flash, full-field (Ganzfeld) electroretinograms (ERGs) recorded from both dark- and light-adapted animals showed no genotype dependence of their peak responses. In contrast to those findings, a more recent study by Laguna et al.^17^ reported significantly increased B-waves, maximum oscillatory potential (OP) amplitudes, and flicker ERG responses in Ts65Dn mice when compared with euploid control mice.^17^ These authors correlated these electrophysiological findings with their histologic observation of a thicker inner nuclear layer (INL) in the retina of Ts65Dn versus control mice.

This study was designed with two main goals in mind. The first one was to expand our knowledge of ocular structures in Ts65Dn and Dp16 mouse models of DS by using a combination of noninvasive and in vitro methods. Accordingly, we examined the cornea and the anterior segment of the eye for the presence of lesions and/or opacification; quantified the thickness of different retinal layers by optical coherence tomography (OCT); analyzed retinal vasculature by bright field and fluorescent imaging and retinal flat-mount preparations; and measured eye and lens dimensions by magnetic resonance imaging (MRI). The second goal was to address the discrepancies between our original ERG results in Ts65Dn mice and those by Laguna et al.,^17^ while producing the first ERG characterization of retinal function in Dp16 mice. Our working hypothesis was that perhaps the different doses of the noncompetitive N-methyl-D-aspartic acid (NMDA) receptor antagonist ketamine used in the general anesthesia protocols in these two studies might have had a significant role in producing the observed discrepancies. This idea was based on the growing body of evidence indicating NMDA receptor dysfunction in Ts65Dn mice.^35^^–^^41^

At the conclusion of this project, we successfully achieved our first goal. Indeed, our new finding of enlarged retinal blood vessel diameters in both Ts65Dn and Dp16 mice may have predicted the finding of such a trait in persons with DS. In addition, we also reconciled some of the discrepancies between the two published ERG studies, mostly by producing evidence that different anesthetic agents (urethane, ketamine, and tribromoethanol) can indeed generate ERGs with different properties. Interestingly, these observed differential effects of anesthetic agents on ERG properties were only seen in control euploid animals.

## Materials and Methods

### Animals

All experimental procedures were approved by the Institutional Animal Care and Use Committee at Case Western Reserve University (CWRU) and were in accordance with the ARVO guidelines for the use of animals in research. Both Ts65Dn and Dp16 mice used in this study were generated by in-house repeated backcrossing (> 10 generations) of either Ts65Dn or Dp16 females (purchased from JAX, Bar Harbor, ME, USA) with B6EiC3SnF1/J hybrid male mice (C57BL/6JEiJ females crossed to C3H/HeSnJ males, also from JAX). Colonies were maintained in a standard 12:12-hour light/dark cycle (lights on at 6:00 AM) in the Animal Resource Center at CWRU. Food and water were available ad libitum. Ts65Dn and Dp16 mice were genotyped using polymerase chain reaction.^29^^,^^30^

In vivo imaging experiments were conducted serially in 161 mice divided into three different age groups: 17-day-old (10 animals per genotype); 35-day-old (10 Dp16 versus 10 wild-type control mice, and 11 Ts65Dn versus 10 euploid control mice); and the “adult group” (22 Ts65Dn versus 18 euploid control mice, and 20 Dp16 versus 20 wild-type control mice; aged 41 to 170 days). Retinal flat-mount preparations were made from 191 mice divided into four age groups: 1-day-old (12 Dp16 versus 12 wild-type control mice, and 9 Ts65Dn versus 13 euploid control mice); 3-day-old (10 Dp16 versus 9 wild-type control mice, and 10 Ts65Dn versus 19 euploid control mice); 6-day-old (10 Dp16 versus 16 wild-type control mice, and 9 Ts65Dn versus 17 euploid control mice); and 9-day-old mice (8 Dp16 versus 12 wild-type control mice, and 8 Ts65Dn versus 17 euploid control mice). ERGs were recorded from 97 mice 3 to 6 months of age (37 Ts65Dn versus 36 euploid control mice, and 12 Dp16 versus 12 wild-type control mice).

### In Vivo Imaging

Mydriasis was produced using drops of topical mydriatic solutions (0.5% tropicamide and 2.5% phenylephrine HCl; Akorn, Lake Forest, IL, USA). Topical lubricant eye gel (Goniovisc 2.5%; Sigma Pharmaceuticals, North Liberty, IA, USA) was applied liberally to maintain the cornea moist and clear during imaging. Mice were anesthetized with 20% urethane (Sigma-Aldrich, St. Louis, MO, USA) in phosphate-buffered saline solution (PBS) (8.0 mL/kg injected intraperitoneally). Anesthetized mice were then placed on an imaging platform and images of both eyes were captured.

#### Slit Lamp Biomicroscopy

Cornea and lens integrity of both eyes was examined using an Anterior Segment Slit Lamp Imaging System (Micron IV; Phoenix Research Labs, Pleasanton, CA, USA).

#### OCT

Retinas were examined using real-time, image-guided OCT (Micron IV, Phoenix Research Labs). Anesthetized mice were placed on an imaging platform and OCT scans of the posterior retina were generated simultaneously with real-time bright-field images. The OCT scan consisted of 100 averaged images centered on the optic disc of both eyes. Segmentation of the retinal layers was performed using the InSight software (Phoenix Research Labs). OCT images were segmented into the following layers: inner limiting membrane (ILM), retinal nerve fiber ganglion cell (RNFGC) complex, inner plexiform layer (IPL), outer plexiform layer (OPL), external limiting membrane, and retinal pigmentation epithelium (RPE). According to layer segmentations, thicknesses were measured for the inner retina (extending from the ILM to the OPL layers), the RNFGC complex (which combines the retinal nerve fiber layer (RNFL) and the ganglion cell layer [GCL]), the IPL (delimited by the RNFGC complex and the upper boundary of the INL), the INL (defined as the layer bordered by the lower boundary of the IPL and the OPL), the RNFGC complex + IPL (which combines the RNFGC complex and the IPL), the outer retina (delimited by the OPL and the RPE layers), and the total retina (defined as the layer bordered by the ILM and the RPE). The thicknesses of retinal layers were obtained by measurements of four lateral distances from the optic disc on both sides (100, 200, 300, and 400 µm).

#### Bright-field Imaging and Fluorescein Angiography

Retinal fundus images of the mouse eyes were obtained using a Micron IV Retinal Imaging Microscope (Phoenix Research Labs), according to anesthesia and mydriasis protocols described above. Anesthetized mice were placed on an imaging platform, and bright-field fundus images (centered on the optic disc) were obtained from both eyes. Next, mice were intraperitoneally injected with 10% fluorescein sodium (AK-Fluor; Akorn) at 5 µL/g body weight, and fluorescent fundus images of both eyes were acquired. The image with the highest quality was selected for further analysis. A specialized computer software (Singapore I Vessel Assessment [SIVA] Analysis, version 3.0, National University of Singapore, Singapore) was used to assess a variety of retinal vasculature parameters (total number of vessels, vessel caliber, vessel fractal dimension, and vessel curvature tortuosity) as previously described by Cheung et al.,^42^ with a trained grader responsible for analyzing the automated SIVA measurements and for manually adjusting any inaccuracy. The measured region was defined as the area from 0.5 to 2.0 disc diameters away from the margin of the optic disc (extended zone).

#### MRI

Left eyes of urethane-anesthetized mice were imaged subsequent to ERG recording sessions of cone function in right eye. Each mouse was positioned on a homemade MRI-compatible holder and allowed to breathe spontaneously during the experiment. Respiration and rectal temperature were continuously monitored, and temperature was regulated using a Model 1025T Monitoring System (Small Animal Instruments, Inc., Stony Brook, NY, USA). MRI data were acquired on a BioSpin 9.4T MRI System (Bruker, Ettlingen, Germany) at the Case Center for Imaging (CWRU, Cleveland, OH, USA) using a 35-mm mouse 1H Volume Coil (RAPID Biomedical GmbH, Rimpar, Germany). Quick coronal, axial and sagittal scans were acquired to center and orient the high-resolution coronal scans on the orbit of the eye and with the two eyes aligned in the transverse direction on both axial and coronal views. Images were acquired using a rapid acquisition with relaxation enhancement protocol. (More methodologic details and results of these experiments can be found in Supplementary Material).

### Retinal Flat-mount Preparation

Retinal flat-mounts were prepared according to Tual-Chalot et al.^43^ with minor modifications. Briefly, postnatal mice on days 1, 3, 6, and 9 were decapitated, both eyes were enucleated and fixed in 4% paraformaldehyde in twice-concentrated phosphate-buffered saline (2X PBS) for 15 minutes at room temperature. Eyes were then washed in cold 2X PBS, and the retinas were dissected from the eyecup, flattened under a stereoscopic microscope, and incubated in cold methanol until proceeding with immunostaining. Dissected retinas were incubated with blocking buffer (PBS + 0.2% bovine serum albumin + 0.3% Triton X-100) for 1 hour at room temperature, followed by incubation with Isolectin IB4 from *Griffonia simplicifolia* Alexa Fluor 488 Conjugate (Invitrogen, Eugene, OR, USA) (1:200 dilution in blocking buffer) at 4°C overnight. On the next day, the retinas were washed four times in PBS + 0.3% Triton (10 minutes each washing) and flat mounted on slides with ProLong Gold Antifade Mountant (Molecular Probes, Eugene, OR, USA). Retinal flat-mount images were captured by immunofluorescence using the EVOS FL Auto 2 Imaging System (Invitrogen, Carlsbad, CA, USA).

Whole retina photographs were generated from multiple images acquired by a tile scan of the entire sample. Adobe Photoshop was used to combine a group of multiple overlapping images into a single composite view. Total vessel length and lacunarity were analyzed by the AngioTool software (NIH National Cancer Institute),^44^ whereas vascular, avascular, and total retinal areas from the retinal flat-mounts were analyzed using ImageJ (National Institutes of Health, Bethesda, MD, USA). When both retinas were available, a mean value for each animal was obtained by averaging the retinal parameters calculated from each mouse retina.

Measurements of arteriole and venule diameters were made from high resolution stitched microscope retinal images. In Adobe Photoshop, an overlay layer containing the reference circle diameter (1.72 mm at P6, 2.05 mm at P9) was visually centered over the retinal vessels. The approximate center-line of each vessel was manually traced in the overlay layer using a unique color for each arteriole and each venule to mark only the section of each vessel that could be readily measured and only the main branch central to any major bifurcation. The original microscope images and the overlay layers were then imported into Wavemetrics IgorPro (WaveMetrics, Inc., Portland, OR, USA) for analysis. The vessel overlay lines were thinned to a single pixel thickness, and also thickened to generously cover the vessels. Histograms of the image brightness under the thickened sections were used to adjust brightness levels. The thinned lines were used to automatically trace the vessels. The tangent to each line was used to extract the image brightness in slices perpendicular to the vessel. All slices within the reference circle for each vessel were ensemble averaged, resulting in a central tendency measure of the vessel's width, while diluting the random effects of the side branches. The width in pixels of the ensemble average for each vessel at the mid-brightness point was extracted and converted to microns. For each retina, average width and count of venules and arterioles were calculated separately. If two retinal preparations were available for a particular mouse, the mean values of these measures were used in statistical analyses.

### ERG

Adult male (3 to 6 months old) Ts65Dn and Dp16 mice and their euploid controls were used in this study. To assess rod function, mice were dark adapted for 12 hours before testing, and all ERGs were recorded in the dark with the aid of a red light. A second group of Ts65Dn, Dp16, and control mice was light-adapted to test cone function in right eye followed by morphometric MRI in the left eye (Supplementary Material). Mice were anesthetized by one of the following three methods: (1) ketamine and xylazine (95 mg/kg and 5 mg/kg, respectively, as a single intraperitoneal injection of 6.25 ml/kg); (2) urethane (20% w/w, intraperitoneal injection at 8.0 ml/kg); or (3) tribromoethanol (TBE; 220 mg/kg, intraperitoneal injection at 6.25 mL/kg). The pupils of mice were dilated with tropicamide 0.5% and phenylephrine 2.5% eye drops, and the cornea was moistened with Goniovisc 2.5%. Mice were then placed in the ERG system (Phoenix Ganzfeld ERG; Phoenix Research Labs), and temperature was maintained with a heating pad (Phoenix Research Labs). Electrical contact with the cornea was achieved by a gold-plated objective lens through which light passed at the tip of the equipment's Maxwellian lens. The reference needle electrode was placed subdermally between the eyes and the ground electrode was placed on the tail. Triggering of the light stimulus and acquisition of the corneal voltage trace were accomplished with the LabScribe2 software through the Phoenix Research Labs ERG module. ERGs were evoked by flashes of green light (504 nm; 1-ms flash duration). Dark-adapted mice were presented 19 different stimulus intensities, ranging from −4.7 to 1.0 log cd.s/m^2^, in increments of 0.3 log cd.s/m^2^. For stimulus intensities varying from −3.8 to −2.0 log cd.s/m^2^, the interstimulus interval was 0.7 s; and for stimulus intensities −1.7 to 1.0 log cd.s/m^2^, the interstimulus interval was 10 s. Mice in the light-adapted protocol were subjected to nine different stimulus intensities, ranging from −0.2 to 2.2 log cd.s/m^2^, in increments of 0.3 log cd.s/m^2^ (interstimulus interval of 10 s). Depending on the stimulus intensity, the response to five to 20 flashes was averaged offline for analysis of waveform characteristics in MATLAB (R2019b; Mathworks, Natick, MA, USA). This analysis consisted of establishing the amplitudes of the A-wave, B-wave, and OPs. The amplitude of the A-wave was measured from the prestimulus baseline to the most negative trough of the ERG, whereas the amplitude of the B-wave was measured from the trough of the A-wave to the most positive peak of the ERG that followed the B-wave. Peak times were measured from flash onset to the peak of the A- and B-waves. Rod-mediated responses were also analyzed using a modified Lamb-Pugh model^45^^–^^48^ (Igor Pro) of the activation phase of the A-wave by fitting to the leading edges of ERGs recorded for flash intensities from –1.1 to 2.2 log cd.s/m^2^ (Supplementary Material Equation 2). According to this model, R_max_ (µV) is the maximum A-wave response, t_c_ (ms) is a characteristic time constant of transduction, and t_d_ (ms) is the delay contained in the response.^45^^,^^46^ During fitting, R_max_ was fixed at the maximal A-wave amplitude recorded, and t_c_ and t_d_ were allowed to vary.

For weak stimuli (i.e., does not produce a significant A-wave) the dark-adapted B-wave amplitude-intensity relation can be described as linear. However, at stronger stimuli, it has been shown that the A-wave will continue to grow whereas the B-wave will saturate.^49^^–^^51^ In our study, this phenomenon was more pronounced in ketamine- and TBE-anesthetized mice than in urethane-anesthetized mice. To describe the saturation of the B-wave amplitude-energy relation beyond its linear range we fit the data with a Naka-Rushton function. The Naka-Rushton function^47^^,^^48^^,^^52^ was fitted to both the light- and dark-adapted B-wave for flash intensities −3.8 to 1.0 log cd s/m^2^ to derive the maximum B-wave amplitude (Vmax [µV]) and the flash intensity that produces the half-maximum response, K (log cd.s/m^2^) (Prism v7.0; GraphPad Software, La Jolla, CA, USA). A fast Fourier transform algorithm implemented in MATLAB (Mathworks) was used to separate the OP from the ERG (Supplementary Material and Supplementary Figure S1). The OP magnitude was measured from the most positive peak to the most negative peak.

### Statistical Analysis

Results are presented as mean ± SEM. Statistica Academic (Statistica, version 13; TIBCO Software, Palo Alto, CA, USA) was used for all the statistical analyses. Mean values of retinal thickness, which were obtained by measurements of multiple lateral distances (dependent variable), were compared by two-way repeated measures analysis of variance (RM ANOVA), with genotype and age as categorical factors. Retinal vascular parameters were also compared using two-way RM ANOVA, with genotype and age as categorical factors. Mean values of ERG peak amplitude, which were measured under multiple stimulus-intensities (dependent variable), were compared by one or two-way RM ANOVA, with genotype or genotype and anesthetic agent as categorical factors. B-wave amplitudes were fitted with the Naka-Rushton function and comparisons between genotypes and anesthetic agents were performed with a sum-of-squares F test in Prism (GraphPad) (see Supplementary Tables S14, S19, and S22 for statistical analysis). Fisher's least significant difference post hoc analysis was performed when genotype-dependence was detected by ANOVA. A *P* value < 0.05 was considered statistically significant. In all figures, *P* values of <0.05, 0.01, and 0.001 were represented as the symbols *, **, and ***, respectively (for simplicity, only *P* values were reported in the text, F statistics can be found in the Supplementary Tables corresponding to a particular section of Results).

## Results

### Morphology and Light Transmission Properties of Lens and Cornea are Preserved in Ts65Dn and Dp16 Mice

**Figure 1.**
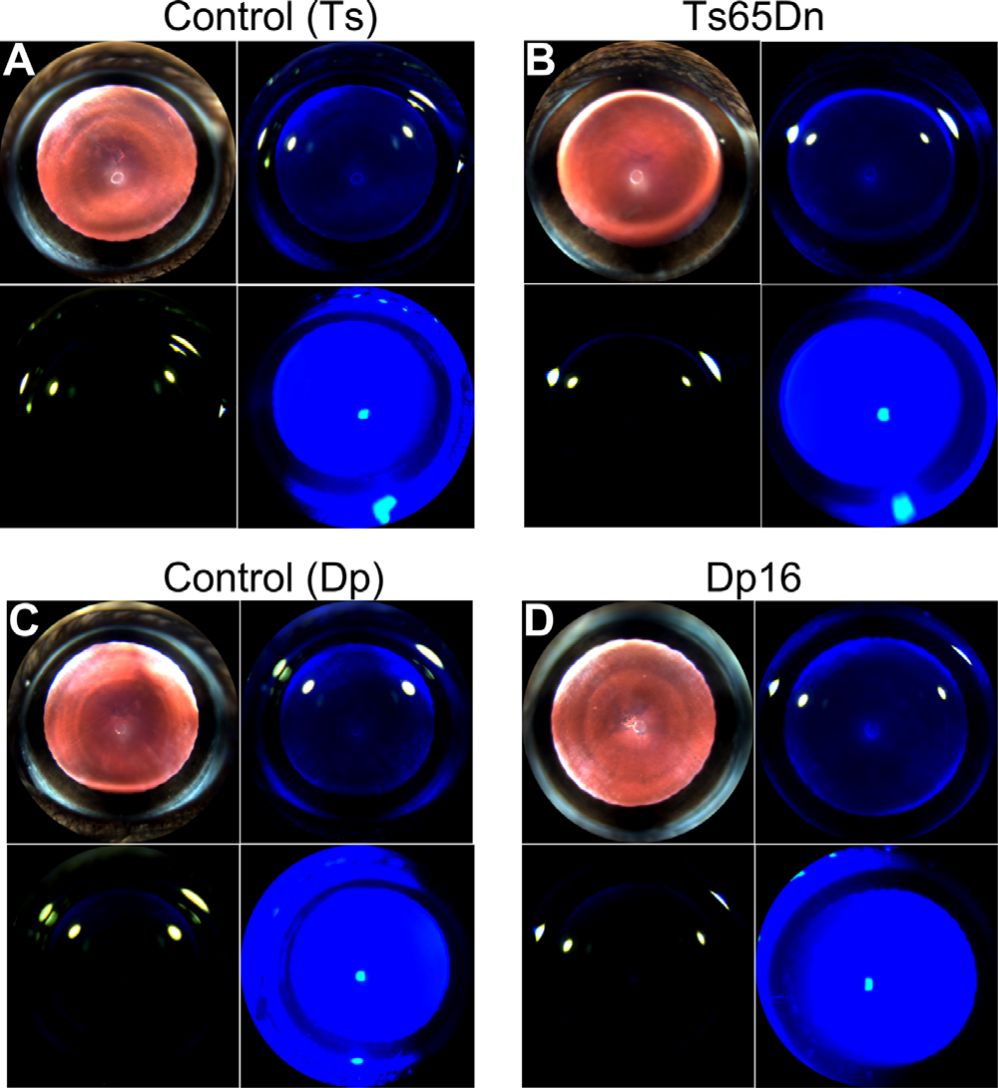
Lens and corneal morphology are preserved in Ts65Dn and Dp16 mice. Representative slit lamp images of (**A**) Control (Ts), (**B**) Ts65Dn, (**C**) Control (Dp), and (**D**) Dp16 mice at 35 days of age.

Slit lamp examination was used to probe for any evidence of congenital or age-related cataracts in the eyes of Ts65Dn, Dp16 mice, and their respective controls. Similar to euploid control mice, neither Ts65Dn nor Dp16 mice showed any signs of congenital or age-related cataracts, which are reported to occur at an increased rate in persons with DS (2%–6%, for congenital cataracts, and 11–60%, for acquired cataracts^10^^,^^11^). In this study, Ts65Dn, Dp16, and their control mice (from now on, referred to as control (Ts) and control (Dp), respectively) displayed corneas and lenses without any sign of opacity or ocular abnormalities (Figs. 1A–D) (with the exception of a single Dp16 mouse, which showed a small unilateral corneal scar; data not shown).

### Both Ts65Dn and Dp16 Mice Display Significantly Increased Inner Retina Thickness

**Figure 2.**
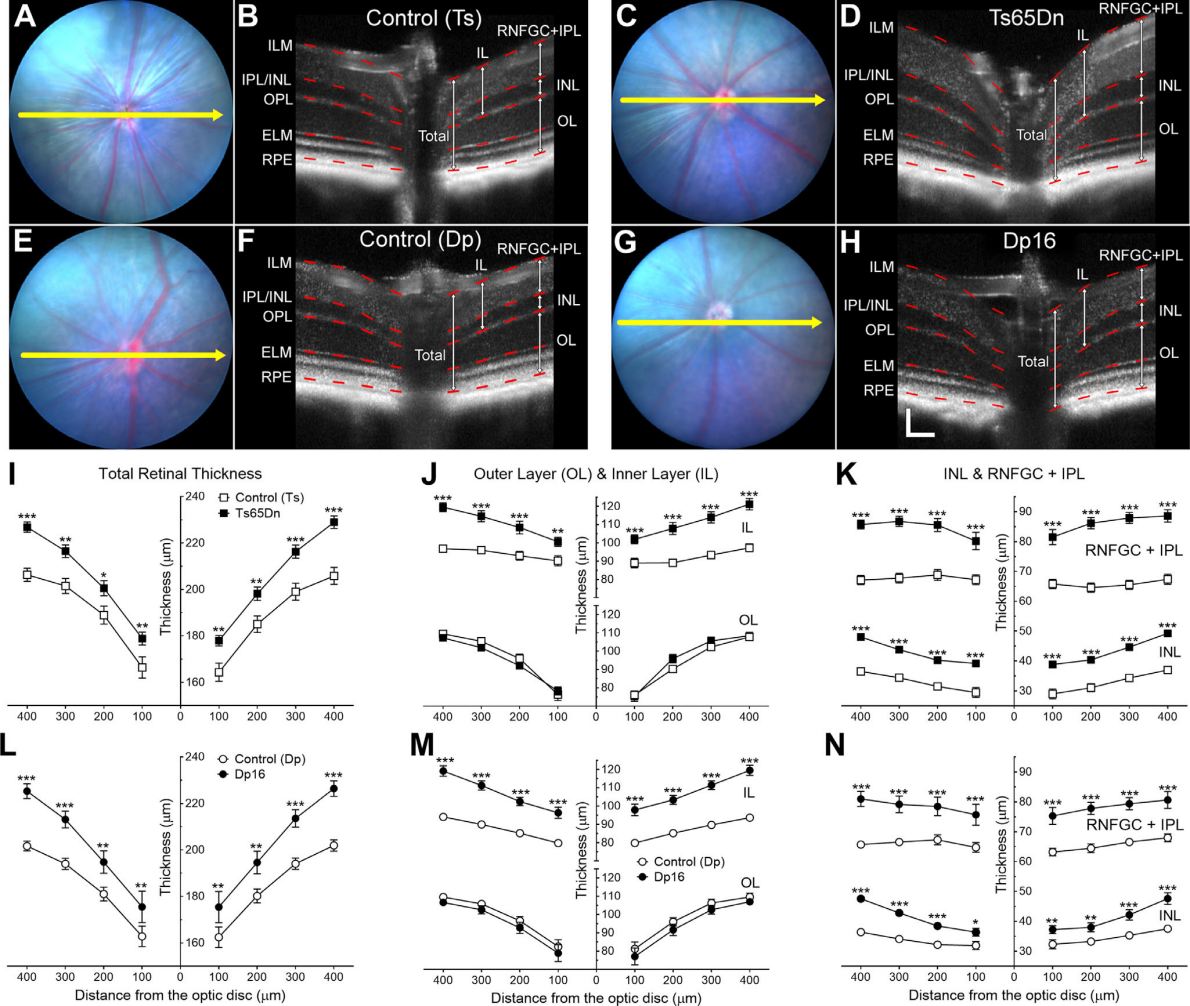
Ts65Dn and Dp16 mice display genotype-dependent increases in total and inner retinal layer (IL) thickness, including the three innermost retinal layers. No genotype effect on the mean thickness of the outer retinal layer (OL) was observed for either chromosomally altered mouse strain. Panels **A, C, E,** and **G** show representative OCT images of retinas from Ts65Dn and Dp16 mice, and their respective controls, at 35 days of age. *Yellow arrows* in bright-field fundus images indicate the position of OCT scans. Panels **B, D, F,** and **H** are examples of OCT scans in which *red bars* within layer boundaries are centered over the 100-, 200-, 300-, and 400-µm distances. (*Calibration bars* in Panel **H** represent 100 µm horizontally and 50 µm vertically.) Retinal layer thicknesses were plotted (mean ± SEM) with four lateral distances from the optic disc on both sides (100, 200, 300, and 400 µm) in panels **I** to **N**. Statistical significance is expressed as *P < 0.05, **P < 0.01, and ***P < 0.001.

Sagittal optical sectioning images of the posterior retina were generated from 100 averaged OCT scans centered on the optic disc (as seen in Figs. 2A–H). Qualitatively, Control (Ts), Ts65Dn, Control (Dp), and Dp16 mice (Figs. 2B, 2D, 2F, and 2H, respectively) all showed typical multilayered organization of retinal morphology.

Quantitative morphometric analyses were performed on the OCT images to obtain mean values of thickness for a total of six retinal layers—the total, outer layer, inner layer and its three innermost layers (retinal nerve fiber ganglion cell [RNFGC] complex plexiform layer, IPL, and INL) —(at 100, 200, 300, and 400 µm, from each side of the optic disc) in 17- and 35-day-old and adult mice of all four genotypes. For simplicity, in this section, we will focus on comparisons between mean retinal thickness values obtained from 35-day-old animals (although age has been included in all analyses as a covariate). The results of these analyses are depicted graphically in Figures 2I–K for Ts65Dn versus control (Ts) mice, and in Figures 2 L–N for Dp16 versus control (Dp) mice. (Results of these same measures for 17-day-old and adult mice were qualitatively and quantitatively similar to those seen in these figures and can be found in Supplementary Figures S2 and S3, respectively.)

Two-way RM ANOVA of total retinal thickness showed that genotype, age, and distance from the optic disc had significant effects on the mean values (*P* < 0.0001, *P* < 0.0001, and *P* < 0.0001, respectively). Significant interactions were observed between distance and genotype (*P* < 0.0001), and between distance and age (*P* < 0.0001). However, no significant interaction was detected between genotype and age (*P* = 0.2347) (Supplementary Table S1). Fisher's post hoc tests confirmed that the observed increase in total retinal thickness was significant for both Ts65Dn and Dp16 mice compared with their respective age-matched control mice in all lateral distances considered (Figs. 2I and 2L; Supplementary Figures S2 and S3 and Tables S1 and S2).

To track the origin of the observed increases in total retinal thickness in the two chromosomally altered mouse models of DS, we performed independent analyses of the mean thickness of the outer and inner retinal layers. As can be inferred from the lower portion of Figures 2J and 2M, we found no significant genotype effect on the mean thickness of the outer retinal layer (*P* = 0.3598). In this analysis, however, the mean thickness of the outer retinal layer was significantly affected by the age of the animals (*P* < 0.0001) and the distance the measurements were made from the optic disc (*P* < 0.0001). In contrast, analysis of the mean inner retinal thickness revealed significant genotype, age, and distance effects on this measure (*P* < 0.0001, *P* < 0.0001, and *P* < 0.0001, respectively). Significant interactions were detected between distance and genotype (*P* < 0.0001), and between distance and age (*P* < 0.0001). However, no significant interactions were observed between genotype and age (*P* = 0.0790), and between distance, genotype, and age (*P* = 0.4829). In addition, post hoc tests identified significant differences between the inner retinal thickness of Ts65Dn and Dp16 mice and their respective age-matched controls for all eight lateral distances assessed (see upper portion of Figs. 2J and 2M, and Supplementary Figures S2 and S3 and Tables S1 and S2).

Finally, we assessed the thickness of the INL (the retinal layer between the inner and outer plexiform layers), which Laguna et al.^17^ has found histologically to be thicker in Ts65Dn mice when compared with euploid control mice, as well as RNFGC complex, IPL, and RNFGC complex and IPL combined. (The boundaries between the RNFL and the GCL have very similar reflectance profiles, which make these boundaries difficult to delineate unequivocally on OCT scans, and, therefore, RNFL and GCL will be combined and analyzed as RNFGC complex here.) Two-way repeated measures ANOVA revealed that genotype, age, and distance had significant effects on mean INL retinal thickness (*P* < 0.0001, *P* < 0.0001, and *P* < 0.0001, respectively). A significant interaction was detected between distance and genotype (*P* < 0.0001), and between distance and age (*P* < 0.0001). An interaction was also observed between genotype and age (*P* = 0.0062), but no significant interaction was observed among distance, genotype, and age (*P* = 0.9945). Fisher's least significant difference post hoc analysis confirmed genotype-dependent differences for both Ts65Dn and Dp16 mice compared with age-matched control mice in all lateral distances (lower portion of Figs. 2K and 2N, and Supplementary Figures S2 and S3 and Tables S1 and S2). In addition, analysis of the mean RNFGC+IPL thickness revealed significant genotype, age, and distance effects on this measure (*P* < 0.0001, *P* < 0.0001, and *P* < 0.0001, respectively). Significant interactions were detected between distance and genotype (*P* < 0.0001), and between distance and age (*P* < 0.0001). However, no significant interactions were observed between genotype and age (*P* = 0.1598), and between distance, genotype, and age (*P* = 0.0802). Post hoc tests identified significant differences between the RNFGC+IPL retinal thickness of Ts65Dn and Dp16 mice and their respective age-matched controls for all eight lateral distances assessed (see upper portion of Figs. 2K and 2N, and Supplementary Figures S2 and S3 and Tables S1 and S2). (The results of retinal thickness analyses of RNFGC complex and IPL were quantitatively similar to those seen in the combined RNFGC+IPL and can be found in Supplementary Tables S1 and S2).

### Ts65Dn and Dp16 Mice Present Mild, but Significant Alterations in Retinal Vasculature

**Figure 3.**
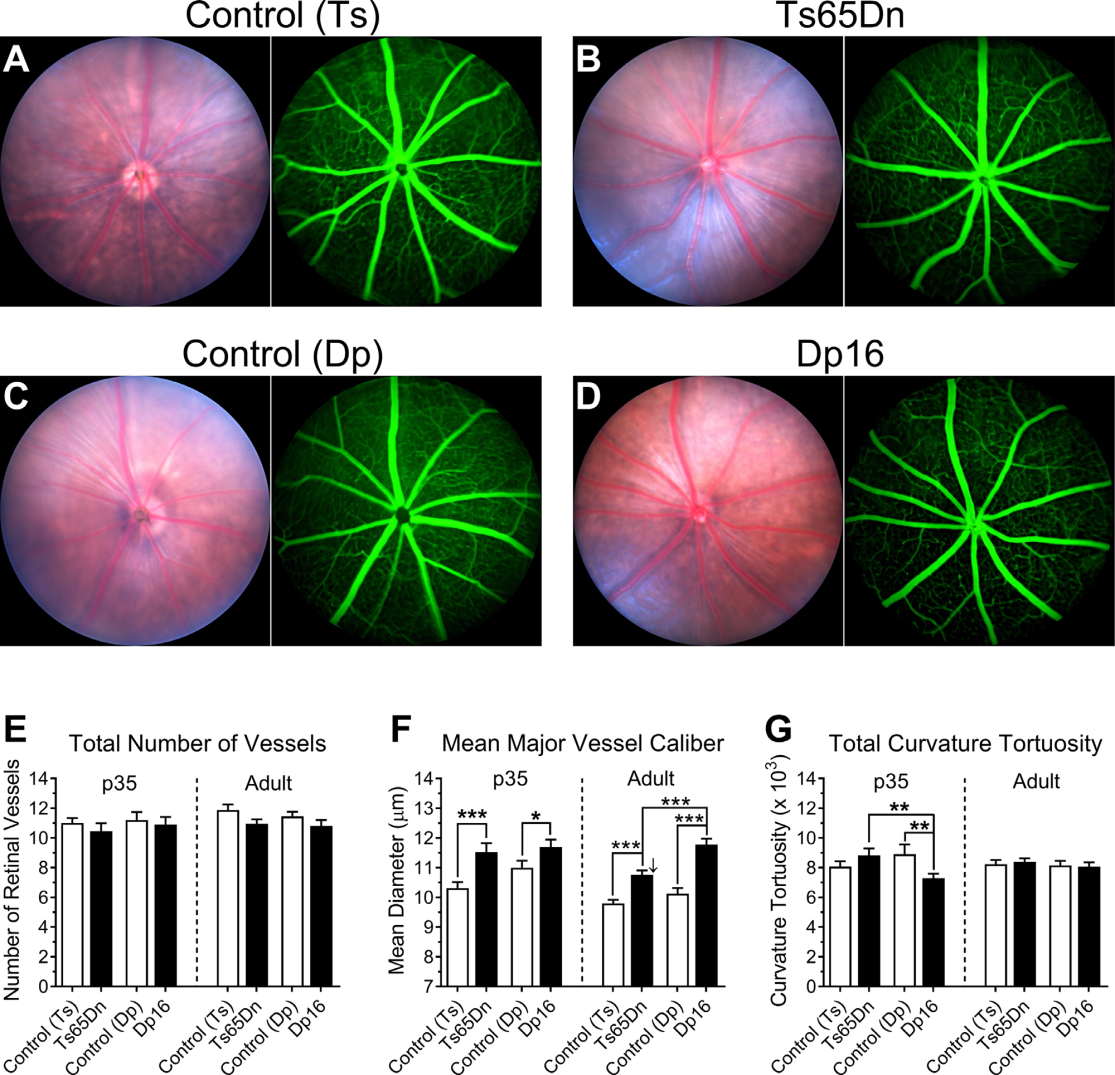
Ts65Dn and Dp16 mice present mild, but significant alterations in retinal vasculature. Qualitatively, bright-field and fluorescein imaging in vivo (panels **A** to **D**) revealed normal retinal vasculature in Ts65Dn and Dp16 mice. No genotype effect on the total number of major vessels was observed for either Ts65Dn or Dp16 mice (**E**). However, significant genotype-dependent effects were found on the caliber of the retinal vasculature for both Ts65Dn and Dp16 mice when compared with their respective age-matched controls (**F**). Post hoc comparisons also revealed a significant reduction in blood vessel caliber in Ts65Dn mice from 35 days of age to full adulthood (*downward pointing arrow*). This age-dependent reduction in blood vessel caliber in Ts65Dn mice resulted in a significant difference in mean retinal vasculature caliber seen between Ts65Dn and Dp16 adult mice. Data represent mean ± SEM. Statistical significance is expressed as *P < 0.05, **P < 0.01, and ***P < 0.001.

Bright-field imaging in vivo was used to assess the fundus of mouse eyes (Figs. 3A–D). Next, mice were injected intraperitoneally with fluorescein, and fundi were imaged for both eyes of 35-day-old and adult mice. (Fluorescein imaging of 17-day-old mice proved technically challenging, and the resulting images were blurred and uneven; therefore these images were not used in the quantitative analyses described here). We focused on the major vessels of the superficial vascular plexus. Qualitatively, we observed normal retinal vasculature, with no evidence of any vasculopathy, in Ts65Dn, Dp16 mice, and their respective controls. Fundus fluorescein angiography revealed normal vessel filling pattern, with no observable vascular leakage of fluorescein to the surrounding areas.

Retinal fundus images were quantitatively analyzed using the SIVA software, which assessed a variety of retinal vasculature parameters. First, we quantified the total number of major vessels, which are defined as the larger vessels that originated from the optic disc center and reached the periphery of the retina. For this measure, RM ANOVA indicated that neither genotype (*P* = 0.1035) nor age (*P* = 0.3019) had any significant effect on the mean of this measure. Also, no significant interaction was observed between genotype and age (*P* = 0.3900) (Fig. 3E). We then analyzed the mean caliber of major retinal vessels. For this particular measure, we found that both genotype and age had significant effects (*P* < 0.0001 and *P* = 0.028, respectively). However, no significant interaction was observed between genotype and age (*P* = 0.1992). Fisher's post hoc tests confirmed significant genotype-dependent differences for the increase in retinal vasculature caliber for both Ts65Dn and Dp16 mice compared with their respective age-matched controls in both mice aged 35 days (*P* = 0.0006 and *P* = 0.0464, respectively) and adult mice (*P* = 0.0017 and *P* = 0.0001; Fig. 3F). Post hoc comparisons also revealed that Ts65Dn mice showed a significant reduction in retinal vasculature caliber from 35 days of age to full adulthood (*P* = 0.0321), whereas a similar phenomenon was not observed in Dp16 mice (*P* = 0.4931). This genotype and age-dependent reduction in blood vessel caliber in Ts65Dn mice resulted in the observation of a significant difference in mean retinal vasculature caliber between Ts65Dn and Dp16 adult mice (*P* = 0.0009), which was not observed at 35 days of age (*P* = 0.6132) (Fig. 3F). The additional retinal vasculature parameters that were quantitatively analyzed using the SIVA software included vascular curvature tortuosity and vasculature fractal dimension. These analyses produced no notable significant genotype-dependent differences, and these results can be found in the Supplementary Tables S3 and S4.

**Figure 4.**
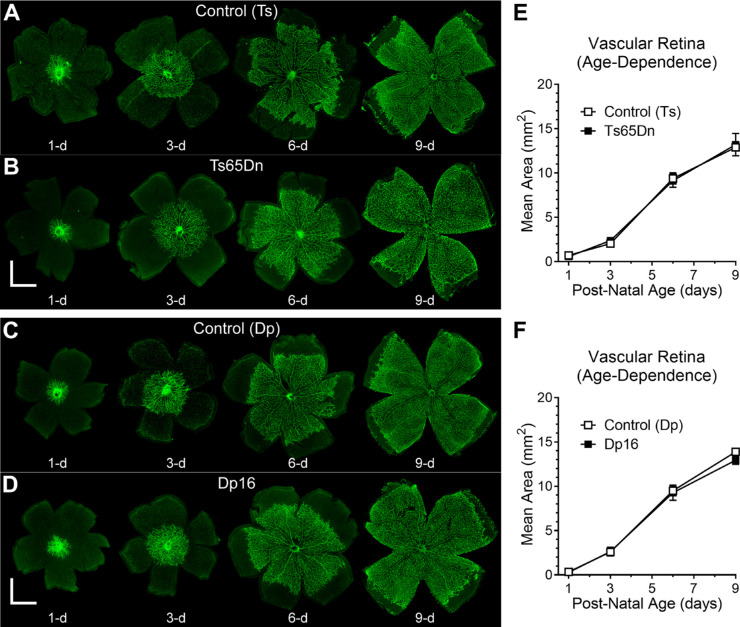
No significant differences in vascularized areas between either Ts65Dn or Dp16 mice and their respective euploid controls were observed during early postnatal development. Panels **A** and **B** show representative images of retinal flat-mounts stained with isolectin-B4 (from postnatal day 1 to 9) in Control (Ts) and Ts65Dn mice, respectively. Panels **C** and **D** depict corresponding representative images for Control (Dp) and Dp16 mice. Panels **E** and **F** represent postnatal expansion of the mean area of the primary plexus from the optic disc toward the periphery of the retina in control (Ts) versus Ts65Dn mice and control (Dp) versus Dp16 mice, respectively. Data points represent mean ± SEM. Statistical significance is expressed as *P < 0.05, **P < 0.01, and ***P < 0.001.

Because of the paucity of phenotypes found in fully developed chromosomally altered mice, we decided to explore the early postnatal development of the primary plexus in these animals in vitro by using retinal flat-mount preparations. To this end, retinas were dissected at postnatal days 1, 3, 6, and 9, incubated in isolectin-B4, and flattened on slides (Figs. 4A–D). We used ImageJ and Angiotool to quantify the age-dependence of total vascular area, total vessel length, and the lacunarity of the vascular network. Once again, we found remarkably little qualitative or quantitative effect of either the Ts65Dn or Dp16 chromosomal alteration on the early development of the retinal vascularization. Figures 4E and 4F provide an example of this. These figures depict the mean vascularized retinal area as a function of postnatal age in Ts65Dn or Dp16 compared with their respective age-matched control mice. RM ANOVA revealed that age (*P* < 0.0001), but not genotype (*P* = 0.3452), had significant effects on this parameter, and no significant interaction was found between genotype and age (*P* = 0.8724). Similar results were obtained for avascularized retinal area, total retinal area, total vessel length, and the lacunarity of the vascular network (see Supplementary Tables S5–S8).

**Figure 5.**
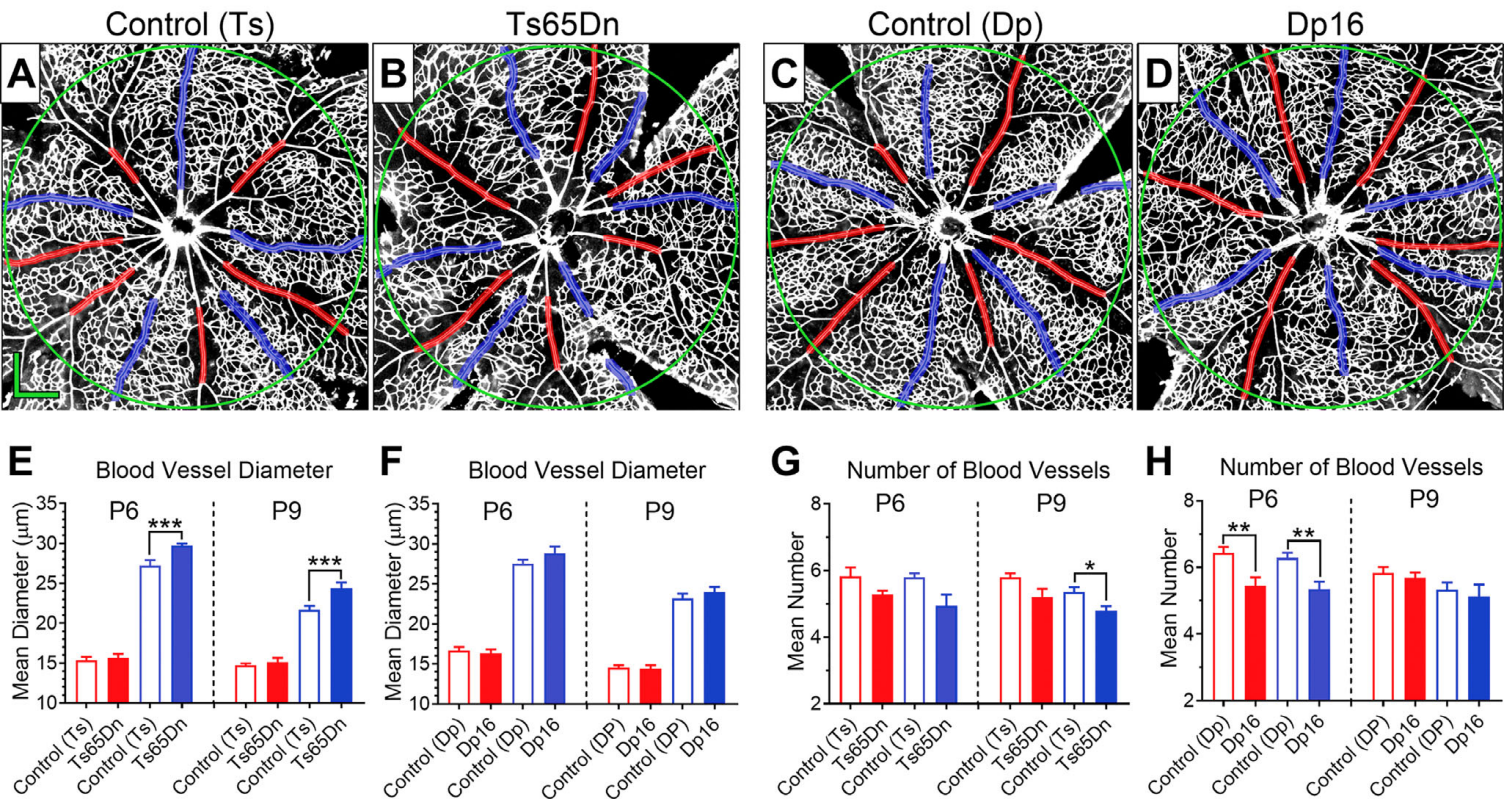
Mild, but significant alterations in retinal vasculature between chromosomally altered mice and their respective euploid controls are present during early postnatal development. Representative high-resolution microscope retinal images processed used in diameter-measurement and counting of arterioles and venules in control (Ts), Ts65Dn, control (Dp), and Dp16 mice (panels **A, B, C,** and **D,** respectively). Blood vessel diameter (**E** and **F**) and number of vessels (**G** and **H**) were quantified from images captured from flat-mount preparations at P6 and P9. Data represent mean ± SEM. Statistical significance is expressed as *P < 0.05, **P < 0.01, and ***P < 0.001.

Given that the mean caliber of major retinal vessels was found to be enlarged in Ts65Dn and Dp16 compared with their euploid control mice, we next quantified the mean caliber and number of major retinal vessels in the microscope images captured from flat-mount preparations at P6 and P9. (At P1 and P3, the arterioles and venules are not well developed enough to allow for precise characterization.) We used a hybrid algorithm developed in our laboratory that consisted of first manually overlaying reference circles and a rough design of the venule and arteriole pathways on these images with Adobe Photoshop, and then further processing these images using an automated script written in IgorPro to precisely delimiting the boundaries of these blood vessels. Figures 5A–D depict typical image outputs from this process. RM ANOVA of the resulting data revealed that genotype (*P* = 0.0046), vessel type (*P* < 0.0001), and postnatal age (*P* < 0.0001) had significant effects on the mean blood vessel diameter. Significant interactions were found between genotype and vessel type (*P* = 0.0445), and between age and vessel type (*P* < 0.0001). However, no significant interaction was found between genotype and age (*P* = 0.9258). Post hoc analysis showed that the diameter of the venules, but not the arterioles in Ts65Dn mice were significantly larger than in Control (Ts) mice at both P6 and P9 (*P* = 0.0008, *P* = 0.0045, respectively; Fig. 5E). No significant differences were found for either arteriole or venule calibers in P6 or P9 in Dp16 mice compared with their controls (Fig. 5F). Regarding the number of major blood vessels, analysis showed that genotype (*P* < 0.0001), vessel type (*P* = 0.0276), and postnatal age (*P* = 0.0066) had significant effects. However, no significant interactions were found between genotype and vessel type (*P* = 0.9840), age and vessel type (*P* = 0.2494), or genotype and age (*P* = 0.1085). Here, post hoc analysis showed no significant differences in the number of venules in P6 or in P9 for Ts65Dn mice (*P* = 0.0664 and *P* = 0.1524, respectively; Fig. 5G). However, the number of arterioles in Ts65Dn mice at P9 (*P* = 0.0209) and arterioles and venules in Dp16 mice at P6 (*P* = 0.0019 and *P* = 0.0083, respectively), were significantly smaller than in euploid control animals, but not at P9 (*P* = 0.6711 and *P* = 0. 5442; Fig. 5H). (See also Supplementary Tables S5, S6, S7, and S8).

### Ts65Dn and Dp16 Mice Display Mildly Altered Scotopic ERGs

**Figure 6.**
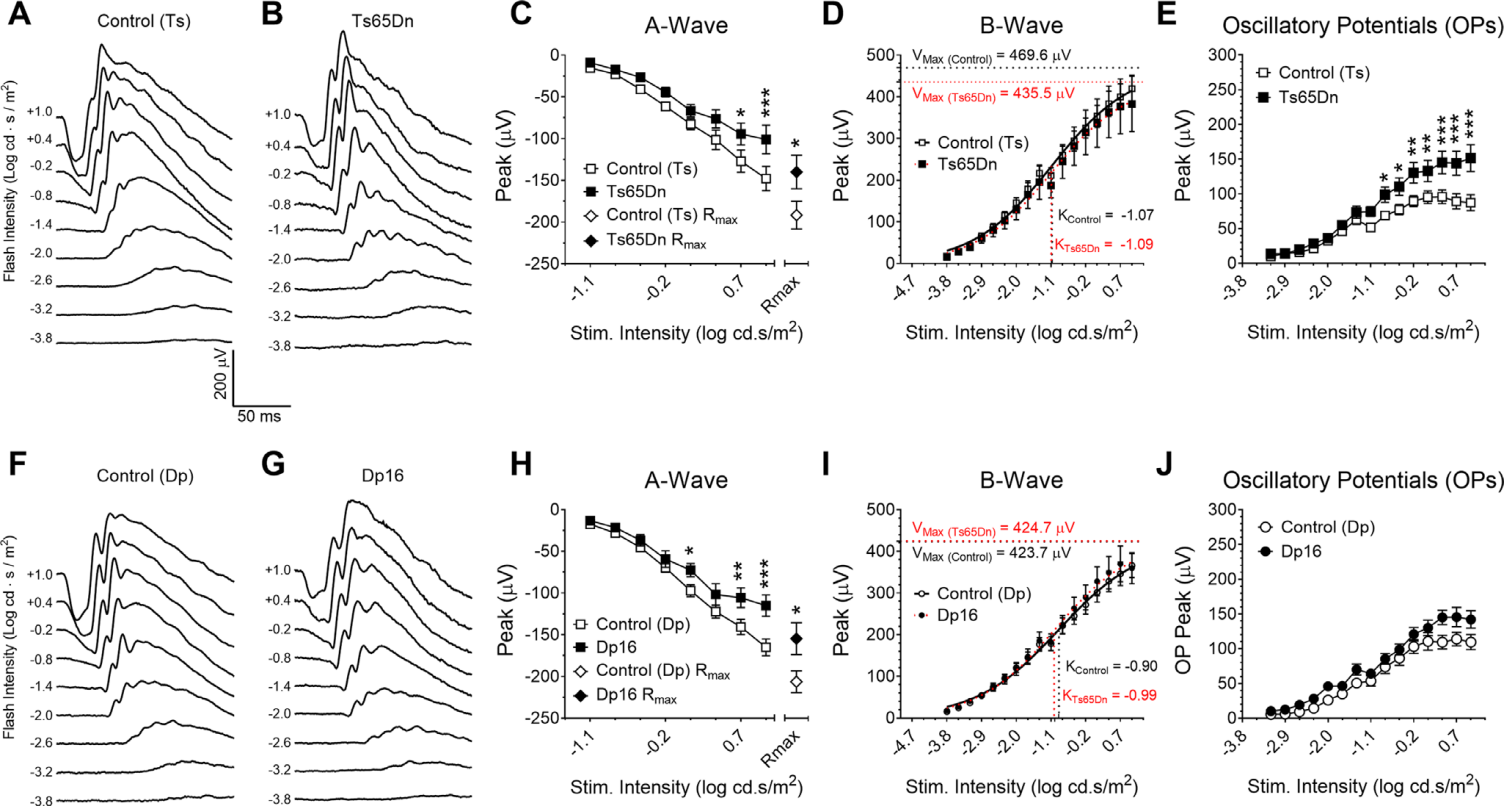
Dark-adapted Ts65Dn and Dp16 mice anesthetized with urethane show a decrease in A-wave amplitude, but no alteration in B-wave, when compared with their respective euploid control mice. Representative traces from (**A**) control (Ts) and (**B**) Ts65Dn mice. (**C**) We observed significantly smaller mean amplitude of the A-wave and maximum rod-mediated response (R_max_) in Ts65Dn mice compared with control (Ts) mice. (**D**) No genotype-dependence was found for the amplitude of the B-wave. The relationship between stimulus intensity and response was modeled using a Naka-Rushton function to derive the maximum B-wave amplitude (V_max_) and the stimulus intensity that produces the half maximum response (K). (**E**) Ts65Dn mice produce larger amplitude OPs when compared with controls. Representative traces from control (Dp) and Dp16 mice (**F** and **G**, respectively). (**H**) Dp16 mice produced smaller mean A-wave amplitudes and R_max_ when compared with control (Dp) mice. However, no genotype dependence was detected for (**I**) B-Wave amplitude, V_max_, K, or (**J**) OP amplitude values. For clarity, only traces for every other stimulus intensity were shown. Mean ERG ± SEM peak amplitudes for euploid control (Ts) (n = 12), Ts65Dn (n = 12), euploid control (Dp) (n = 12) and Dp16 (n = 12) mice under urethane anesthesia. Statistical significance is expressed as *P < 0.05, **P < 0.01, and ***P < 0.001.

The ERG waveform (Figs. 6A–B) consists of an early negative wave (A-wave), followed by a large positive deflection (B-wave). Oscillatory potentials (OPs) are high-frequency, low-amplitude potentials that are often superimposed on the ascending phase of the B-wave of the ERG. To investigate the presence of potential differences in rod-mediated retinal function between dark-adapted Ts65Dn, Dp16, and their respective euploid control mice, we assessed ERG responses to flash stimuli of various intensities. In this set of experiments, mice were anesthetized with urethane. Typical averaged traces are shown in Figs. 6A and 6B for control (Ts), Ts65Dn, and Figs. 6F and 6G control (Dp) and Dp16 mice (for clarity, every other stimulus intensity was shown). Mean peak values for A-wave, R_max_, B-wave, V_max_, K, and OPs as functions of stimulus intensity for Ts65Dn versus control (Ts) mice are depicted in Figs. 6C-6E, and those for Dp16 mice versus control (Dp) are shown in Figs. 6H–J. For the amplitude of the A-wave in these four cohorts of animals, two-way RM ANOVA revealed significant dependences for genotype (*P* = 0.0167) and stimulus-intensity (*P* < 0.0001), and a significant interaction between stimulus intensity and genotype (*P* < 0.0001). Ts65Dn and Dp16 mice also showed significant decreased maximum rod-mediated responses (R_max_) when compared with their respective euploid control mice. For the B-wave, analysis revealed a significant stimulus-intensity dependence (*P* < 0.0001); however, no significant genotype dependence (*P* = 0.71) or significant interaction between stimulus intensity and genotype (*P* = 0.9999) was found. Naka-Rushton analysis did not reveal any significant genotype dependence for V_max_ and K values. Furthermore, additional B-wave analysis, using a physiological activation phase model did not reveal any significant genotype or anesthesia effect. Finally, both genotype (*P* = 0.0156) and stimulus-intensity (*P* < 0.0001) were found to have significant effects on OP amplitude. We also found a significant interaction between stimulus intensity and genotype (*P* < 0.0001). (See also Supplementary Material and Supplementary Tables S9, S12–S15, and S23).

Detailed results of post hoc analyses of the data presented in Figure 6 can be found in Supplementary Tables S10, S11, and S13. Here, it suffices to note that, unlike what was described in Laguna et al.,^17^ we observed a decrease in the mean amplitude of the A-wave at high stimulus intensities, and no differences in B-wave mean amplitude for either Ts65Dn or Dp16 mice in relation to their euploid controls when these mice were anesthetized with urethane. However, similar to what was described by these authors, we observed increased OPs mean amplitude at the higher end of the stimulus intensity in Ts65Dn versus control (Ts) mice (but not in Dp16 versus control (Dp)).

### Scotopic ERG Properties in Control (Wild-Type) Mice are Dependent on the Type of Anesthetic Agent Used in the Experiment

**Figure 7.**
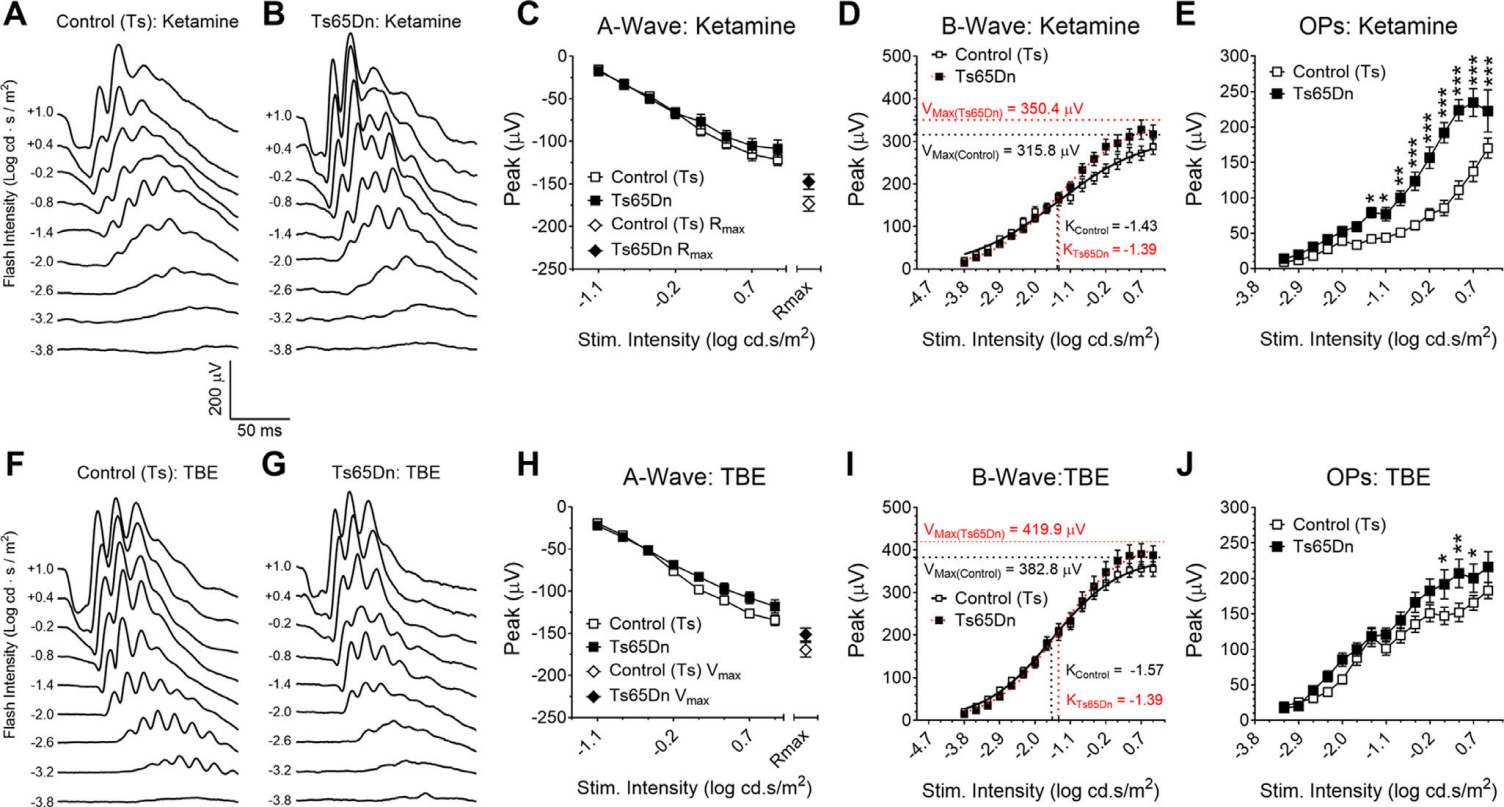
ERG recordings from dark-adapted Ts65Dn mice anesthetized with either ketamine/xylazine or TBE. Representative traces from (**A**) control (Ts) and (**B**) Ts65Dn mice. No genotype-dependence was observed for (**C**) A-wave mean peak amplitude, Rmax, or in (**D**) mean peak amplitude of the B-wave, V_max_, or K. In addition, (**E**) OP amplitudes were not significantly different between Ts65Dn and control (Ts) mice. (**F**) and (**G**) are representative traces from control (Ts) and Ts65Dn mice under TBE anesthesia. Again, no genotype-dependence was observed in A-wave mean peak amplitude, R_max_ (**H**), or (**I**) B-wave mean amplitude, V_max_, or K. (**J**) Ts65Dn produced larger OP mean amplitudes when compared to control (Ts) mice. For clarity, only traces for every other stimulus intensity were shown. Mean ERG ± SEM peak amplitudes for euploid control (Ts) (n = 12) and Ts65Dn (n = 12) mice under ketamine anesthesia, and euploid control (Ts) (n = 12) and Ts65Dn (n = 13) mice under TBE anesthesia. Statistical significance is expressed as *P < 0.05, **P < 0.01, and ***P < 0.001.

Given the previous set of results obtained from mice under urethane anesthesia, and its disagreement with previously published results by others, we decided to test the effects of two additional anesthetic agents on the properties of ERGs recorded from two additional cohorts of Ts65Dn and euploid control mice. In these experiments, dark-adapted mice were anesthetized with ketamine/xylazine (Figs. 7A and 7B) or tribromoethanol (TBE; Figs. 7F and 7G). The results from experiments involving the three separate cohorts of Ts65Dn and euploid control mice, recorded under urethane, ketamine, and TBE, were then analyzed by two-way RM ANOVA. This analysis revealed a significant genotype (*P* = 0.0124) and stimulus-intensity (*P* < 0.0001) dependence for the A-wave, with a significant interaction between these two variables (*P* < 0.0001). Although the effect of anesthetic agent used did not reach significance, the statistics were borderline (F_(2,67)_ = 2.751, *P* = 0.0711), and a significant interaction was detected between anesthetic agent and stimulus intensity (F_(14,469)_ = 1.732, *P* < 0.0465). Such interaction is probably a reflection of the small, but significant difference in mean amplitudes of the A waves recorded from Ts65Dn mice compared with euploid controls under urethane, but not under ketamine or TBE anesthesia at the high-end of the stimulus intensity (Figs. 6C, 7C, and 7H). However, two-way ANOVA did not show a significant difference in maximum rod-mediated response (R_max_) between Ts65Dn and control (Ts) mice for either anesthetic agent (Figs. 6C, 7C, and 7H). (Detailed results of two-way RM ANOVA and post hoc analyses of the data presented in Figure 7 can be found in Supplementary Tables S15 to S19).

Analysis of the mean peak of the B-wave revealed a significant stimulus-intensity dependence (*P* < 0.0001), but no significant dependence on genotype (*P* = 0.8502) or anesthetic agent (*P* = 0.0622). In addition, no interactions were found between anesthetic agent and genotype (*P* = 0.6981) or between stimulus intensity and genotype (*P* = 0.0517) (Figs. 6D, 7D, and 7I). Naka-Rushton function analysis did not show any significant genotype dependence for either V_max_ or K for mice under ketamine or TBE anesthesia. Detailed analysis for the rod-mediated B-wave did not show any significant interaction between anesthetic agent and genotype for mice under ketamine or TBE anesthesia. Finally, analysis of the OP mean amplitudes resulted in significant dependences on genotype (*P* < 0.0001), stimulus-intensity (*P* < 0.0001), and anesthetic agent (*P* < 0.0001). In addition, we found significant interactions between stimulus intensity and genotype (*P* < 0.0001) and between intensity and anesthetic agent (*P* < 0.0001). Although no significant interaction was found between genotype and anesthetic agent (*P* = 0.2379), a triple interaction was found between genotype, anesthetic agent, and stimulus intensity (*P* = 0.0035). In the data depicted in Figs. 6E, 7E, and 7J, one can again see that most of the genotype-dependent differences in OP mean amplitudes occurred at the high end of stimulus intensity. Also, it is important to note that post hoc analyses of the genotype versus anesthetic agent interaction reveal that there was no significant difference in OP mean amplitudes between euploid control mice anesthetized with urethane versus ketamine (*P* = 0.7220), whereas a significant difference was found for this ERG parameter when we compared results from euploid mice anesthetized with ketamine versus TBE (*P* < 0.0001). In contrast, for Ts65Dn mice, we found a significant difference between OP mean amplitudes from animals anesthetized with urethane vs. ketamine (*P* = 0.0222), but no significant difference in this parameter between mice anesthetized with ketamine versus TBE (*P* = 0.1465). (See Supplementary Tables S23, and S24 for complete analysis of the rod-mediated B-wave)

### Photopic ERG Properties Are Not Altered in Ts65Dn and Dp16 Mice

**Figure 8.**
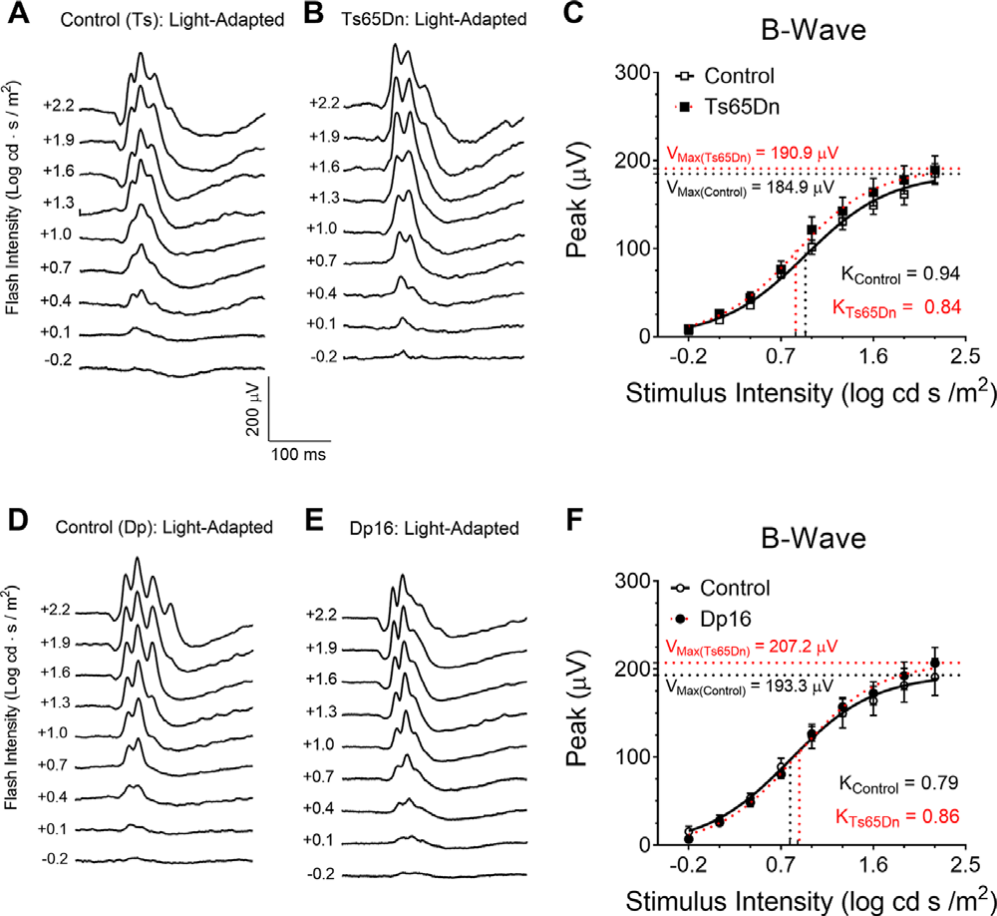
ERG from light-adapted Ts65Dn and Dp16 mice anesthetized with urethane. Representative traces from control (Ts) (**A**) and Ts65Dn mice (**B**). No genotype-dependence was observed for B-wave mean amplitude, V_max_, or K (**C**). Representative traces from control (Dp) (**D**) and Dp16 mice (**E**). Again, no genotype-dependence was observed for B-wave amplitude, V_max_, or K (**F**). Mean ERG ± SEM peak amplitudes for light-adapted euploid control (Ts) (n = 12), Ts65Dn (n = 12), euploid control (Dp) (n = 12) and Dp16 (n = 12) mice.

In another set of experiments, we investigated potential differences in cone-mediated responses between Ts65Dn, Dp16, and their respective euploid control mice. Under urethane anesthesia, we assessed ERG responses to flash stimuli of various intensities. Analysis of the mean peak of the B-wave by two-way RM ANOVA for this group of mice revealed a significant stimulus-intensity dependence (*P* < 0.0001) but no significant dependence on genotype (*P* = 0.430). In addition, no interaction was found between stimulus intensity and genotype (*P* = 0.857) (Fig. 4 C). Similar to what we have seen for Ts65Dn mice, analysis of the mean peak of the B-wave for Dp16 vs Control (Dp) mice revealed a significant stimulus-intensity dependence (*P* < 0.0001), but no significant dependence on genotype (*P* = 0.865) for cone-mediated responses. In addition, no interaction was found between stimulus intensity and genotype (*P* = 0.763) (Fig. 8 F), and Naka-Rushton function analysis of V_max_ and K values did not show significant differences between light-adapted Ts65Dn and control (Ts) mice, or between Dp16 and control (Dp) mice. (See also Supplementary Tables S20 to S22).

### MRI of the Eye Did Not Reveal Any Gross Genotype-Dependent Morphometric Alterations in Ts65Dn and Dp16 Mice

MRI of the left eye were performed sequentially on the same urethane-anesthetized animals used for testing the photopic ERG properties from the right eye described in the previous paragraph, which made these simple experiments very convenient and efficient. However, because we did not have access to a specialized “eye coil” for this study, which would have produced enhanced images, our typical spatial resolution with a standard small-animal coil was limited to 80 µm in the axial and equatorial dimensions. Given that a 5.4- to 6.5-µm change in axial length in the mouse eye corresponds to 1 diopter (D) change in refractive error,^53^ the level of resolution accomplished here limited the detection of genotype-dependent axial lengths alterations to the equivalent of approximately 12 D. With this limitation in mind, using MRI, we have not detected any genotype-dependent alteration on axial length, equatorial distance, or asphericity in Ts65Dn or Dp16 mice when compared with their respective controls (data shown in Supplementary Figure S5 Tables S25 to S28 and Supplementary Material).

## Discussion

This study produced extensive information on ophthalmic parameters in two chromosomally altered mouse models of DS. Some of the observed phenotypes mimicked what has been described in persons with DS, and therefore should help advance the use of these animals in the modeling of this complex genetic disorder. In addition, we found a new quantitative anatomical trait: enlarged retinal blood vessel diameters in both Ts65Dn and Dp16 mice. Such discovery may herald the finding of such phenotype in individuals with DS. The careful assessment of ERG waveform properties also proved to be very informative. For example, we were able to reproduce the observation by a previous study^17^ of an increased amplitude of OPs in Ts65Dn versus their control mice. We also found that one supposedly genotype-dependent ERG feature that had been described previously, an increase in B-wave amplitude in Ts65Dn mice, is likely to be nothing but an artifact of the type of anesthetic agent used during the recording sessions. Additionally, some important DS ophthalmic phenotypes, such as congenital or acquired cataracts, and the occurrence of supranumerary retinal blood vessels, were not observed in the two chromosomally altered mice studied here. Ultimately, it can be argued that perhaps one of the most remarkable findings of this study is how resilient the anatomy, physiology, and postnatal development of the eye and retina can be even in the face of large-scale chromosomal alterations.

One of the DS-like quantitative features we observed was the increased mean total retinal thickness in Ts65Dn and Dp16 mice when compared with their respective euploid control mice. For both chromosomally altered mice, this phenotype was primarily restricted to increases in the thickness of inner retinal layers. Therefore, these findings partially mimic published work by O'Brien et al.,^54^ in which these authors used OCT to compare central subfield thickness between a group of children with DS and an age-matched group of healthy children without DS. Similar to our results, these authors found an increased total retinal thickness in children with DS compared with children without DS in the control group. However, they reported significantly greater thickness of both inner and outer retinal layers in the DS group than the control group. This discrepancy may reflect species-dependent differences, given that the OCT images acquired by O'Brien et al.^54^ were centered on the macula, whereas here we studied mice, which are afoveate animals, and we centered our images on the optic nerve.

A conceivable shortcoming of using OCT to compare retinal-layer thickness between individuals with and without DS is the potential for measurement errors due to possible differences in eye axial lengths.^55^^,^^56^ In the work of O'Brien et al.,^54^ although these authors did not perform direct axial length measurements in their study, they included hypermetropes in their control group and still observed thicker retinas in their group with DS. It should also be noted that there was no significant difference in refractive errors between the two groups (*P* = 0.395, on a two-way *t*-test performed on the published data).

The MRI-based measurements performed in the present study did not reveal any significant difference in axial length between the eyes of chromosomally altered mice and their respective euploid controls. The limited resolution of these measurements (80 µm), however, prevented us from completely discounting the possibility of systematic errors in the assessment of retinal layer thickness. Still, these MRI-based measurements also provided an upper limit to any such potential errors. It is also important to notice, that the observed differences in inner retinal layers between chromosomally altered mice and their respective euploid control mice reported here were all highly significant and consistent across the lateral dimension. Additionally, the specific use of the OCT equipment used in this study has been recently validated by the work by Mishra et al.,^57^ who studied a streptozotocin-induced mouse model of diabetic retinopathy. In that work, these authors found comparable results when investigating the effect of overexpression of the gene *Sirt1* on retinal layer thickness in these mice by using both OCT-based and histology-based measurements. Finally, the OCT anatomic findings from the present study also reproduced the published work by Laguna et al.,^17^ who found significant increases in the thickness of the INL and IPL using histologic sections of the retina from Ts65Dn mice compared with those from control euploid animals.

This study significantly expanded the previous published findings by showing that the increased thickness of the inner retinal layers in Ts65Dn mice can be detected from as early as 17 days of postnatal age until well into adulthood. Furthermore, we report here for the first time that the same phenomenon is also present in Dp16 mice, which shows the robustness of this DS-like phenotype across two different chromosomally altered mouse models of DS. Given that the chromosomal duplication seen in Dp16 mice also includes the *Dirk1A*^31^ gene, our results also indirectly support the previously proposed molecular hypothesis.^17^

We also performed a comprehensive analysis of the postnatal development of the retinal primary vascular plexus, as well as a bird's-eye examination of retinal blood vessels in fully developed retinas of both Ts65Dn and Dp16 mice. One of the initial motivations behind our investigation of retinal vasculature in Ts65Dn and Dp16 mice was to probe whether these animals recapitulate the classical observation of an increased number of major retinal vessels in individuals with DS.^18^^–^^20^^,^^22^^,^^23^^,^^58^^,^^59^ It has been proposed that such increased number of retinal vessels in persons with DS might be related to an early branching of the retinal vessels,^60^ which has been hypothesized to result from a systemic reduction in angiogenesis due to high circulating levels of endostatin.^60^^,^^61^ Endostatin is an antiangiogenic factor produced by the proteolytic cleavage of the matrix protein collagen XVIII, which is encoded by the human chromosome 21 gene *COL18A1*. However, it is important to note that the mouse orthologue of the *COL18A1* gene, *Col18a1,* is located in mouse chromosome 10 and therefore is not present in three copies on either Ts65Dn or Dp16 mice.

Although the vascular phenotypes identified here were generally mild, they are potentially consequential in the interpretation of previous ophthalmic findings associated with DS. For example, we found evidence a reduced (instead of increased) number of retinal venules early in postnatal development (detected at P6). We know that the early postnatal retinal vascular development in mice proceeds in a very tightly regulated pattern.^62^^,^^63^ Consequently, this process has not only been considered as a suitable model to assess physiologic and pathologic development of retinal angiogenesis, but also as a general indicator of the angiogenic process in many other tissues. In general, the primary vascular plexus arises from the center of the optic disc at birth, and grows outward progressively by radial expansion toward the periphery of the retina, reaching the edge by day 7 to 10.^62^^,^^63^ We found that the early postnatal growth of the primary plexus in terms of its overall area was not significantly affected in either Ts65Dn or Dp16 mice. Because we used flat mount preparations at early postnatal stages, which is a technique that allows one to easily identify venules from arterioles, these experiments also helped us to identify enlarged retinal venule diameters in Ts65Dn mice as early as P6. This alteration in mean venule diameter may be the same alteration identified in vivo in both Ts65Dn and Dp16 mice through the use of fluorescein angiography in animals at P35 and older. And it may also reflect the possibility that a thicker retina could lead to increased metabolic demand and altered blood supply.

In the first set of ERG experiments, animals were anesthetized with urethane, which has modest effects on multiple neurotransmitter receptors. According to a 2002 study by Hara and Harris,^64^ at concentrations close to the anesthetic EC50, urethane enhances the functions of α_1_β_2_γ_2S_ GABA_A_ and α_1_ glycine receptors by 23% and 33%, respectively. (For comparison, TBE, at its EC20, enhances α_2_β_1_ GABA_A_ and α_1_ glycine responses by more than 100%.^65^) At this same dose, urethane inhibits the functions of GluN1a/GluN2A NMDA and GluA1/ GluA2 AMPA receptors by 10% and 18%, respectively. Because of its modest effects on these major neurotransmitter receptors, Hara and Harris^64^ argue that urethane should be suitable for maintaining anesthesia during electrophysiological recordings. Accordingly, the results of these ERG experiments with urethane were in agreement with our first study,^34^ that is, we did not observe any increase in the mean amplitude of the B-wave in Ts65Dn mice compared with its euploid controls. Instead, we saw a decrease in the mean amplitude of the A-wave in Ts65Dn mice when compared with controls.

We then recorded ERGs from mice under the same dose of ketamine used by Laguna et al.^17^ Ketamine is a NMDA receptor noncompetitive antagonist and reduces NMDA receptor function more than 80% at its anesthetic EC50, but it has no effect on GABA_A_, glycine, or AMPA receptors.^66^^,^^67^ But even with ketamine anesthesia, analysis of the resulting data failed to detect a genotype-dependent increase in mean B-wave amplitude. Additionally, we found that both Ts65Dn and Dp16 mice anesthetized with urethane display smaller-amplitude A-Waves then its euploid controls, which did not occur for Ts65Dn mice anesthetized with ketamine. Interestingly, this was a consequence of the fact that the mean amplitude of A-Waves in control animals was smaller when they were anesthetized with ketamine versus urethane, whereas the mean amplitude of this parameter did not change in Ts65Dn mice. This was a surprising finding for us, given the previously mentioned growing evidence of NMDA receptor dysfunction in Ts65Dn mice.^35^^–^^41^ Still, these results, in which the ERG properties in wild-type mice was dependent on the anesthetic used, are a reminder that consideration should be given to the choice of anesthetic agents in such experiments. Finally, we did not detect any genotype-dependency for the B-wave in either Ts65Dn or Dp16 mice when anesthetized with urethane. Because Dp16 mice display the same level of thickening of the inner retinal layers as Ts65Dn mice, this finding should be considered as strong evidence against the hypothesized connection between large-amplitude B-waves and thicker inner retinal layers.

The finding of increased retinal vessel calibers in both Ts65Dn and Dp16 mice may be related to a decrease in vascular basal tone or impaired vascular smooth muscle contractility in these chromosomally altered mouse models of DS. Perhaps it may also model a similar phenotype in individuals with DS. Such hypothesis is supported by the fact that individuals with DS are significantly hypotensive when compared with the general population,^68^^–^^70^ as well as the observation that coronary or peripheral vascular disease occurs less frequently in this population when compared with persons without DS.^71^^–^^73^ Therefore, if the retinal vascular phenotype we found in the two chromosomally altered mouse models of DS studied here can be replicated in persons with DS, the study of retinal vasculature could provide a window into the investigation of the effect of trisomy 21 on systemic vascular physiology.

Finally, in the complex three-dimensional topologic organization of the peripapillary vascular plexuses,^62^^,^^63^^,^^74^ it is possible that the observed thickening of the inner retinal layers in Ts65Dn and Dp16 mice is a reflection of larger-diameter blood vessels in the intermediate capillary plexus (located at the inner boundary of the INL) and/or the deep capillary plexus (located at the outer boundary of the INL). Unfortunately, the methodology used in the present study is insufficient to address these issues. Perhaps future studies, using for example confocal microscopy of perfusion labeled flat-mounted mouse and human retinas to reconstruct three-dimensionally retinal microcirculation, such as recently described by Chandrasekera et al.,^74^ may eventually offer satisfactory answers to these questions.

## Supplementary Material

Supplement 1
