# Supplementary material for: Quantitative Analysis of Retinal Structure and Function in Two Chromosomally Altered Mouse Models of Down Syndrome
Source: Invest Ophthalmol Vis Sci. 2020 May 16;61(5):25. doi: 10.1167/iovs.61.5.25 (PMC7405684; doi:10.1167/iovs.61.5.25)
Supplement: Supplement 1 [file iovs-61-5-25_s001.pdf]

Supplementary Material for:

Quantitative analysis of retinal structure and function in two chromosomally altered mouse models of Down syndrome

Authors: Daniella B. Victorino[1, 2], Jonah J. Scott-McKean[1], Mark W. Johnson[1], Alberto C. S. Costa[1, 3]

Affiliations:
1) Division of Pediatric Neurology, Department of Pediatrics, Case Western Reserve University, Cleveland, OH, United States.
2) Postgraduate Program in Neurology and Neuroscience, Federal University of São Paulo, São Paulo, SP, Brazil.
3) Department of Psychiatry, Case Western Reserve University, Cleveland, OH, United States.

SUPPLEMENTARY MATERIALS AND METHODS

Magnetic resonance imaging (MRI):

Images were acquired using a Rapid Acquisition with Relaxation Enhancement (RARE) protocol (aka fast or turbo spin echo). MRI sequences were optimized over the course of the study, with the following final parameters (and ranges): repetition time=4000ms (2000-4000); turbo factor=4x (1-8); echo interval=8ms; effective echo time=16ms (8-24); in-plane resolution=80x80μm; slice thickness=640μm (400-800); repetitions=2 (1-5); matrix size=256x192 (or 256x256); and scan time=6:24 min:sec (3:12 to 12:48). Additional scans were acquired at slice offsets of 1/2, 1/4, and 3/4 of the thickness, when time permitted. The geometry of the eye was measured by fitting two four-part sigmoidal curves to the image intensity along axial and trans-equatorial cross-sections through the center of the eye within the coronal MRI slice most closely centered on the globe. To improve signal to noise ratio, at each point along each cross-section, seven pixels along the orthogonal axis were averaged. The sigmoidal curves were fitted successively and jointly from the center outward, fitting the transition from the hypo-intense lens outward to the hyper-intense anterior and vitreous chambers first, then additive sigmoidal curves were fitted from the chambers to the hypo-intense sclera. The axial length (AL) and equatorial distance (ED) measures of the globe were taken as the distance between the hypo-intense edge just outside the anterior and vitreous chambers. The lens axial length (LAL) and lens equatorial distance (LED) were taken as the distance between each inner pair of sigmoids at the mid-intensity (50%) point along the continuous curves. The asphericity of the eye and lens were calculated as the ratio of axial/equatorial dimensions. The inter-eye distance was the measured from the center of the left eye to the center of the right eye on the MRI slice, which, together with weight, were used to assess whether eye dimensions were related to mouse size (Supplementary Figure 5 and Tables S25-S28).

Electroretinography (ERG): Separation of the Oscillatory Potentials (OPs)

To analyze scotopic (dark-adapted) ERG responses from Ts65Dn or Dp16 and control mice, we used MATLAB R2017b for offline separation of the oscillatory potentials (OPs) from the other wave components. This was done by applying a fast Fourier transform (FFT) to obtain the frequency spectrum of the ERG responses (Supplementary Figure S1). ERGs displayed two distinct amplitude regions in the frequency domain. The low-frequency region reflected the A- and B-waves (Supplementary Figure S1C) and the high-frequency region reflected the OPs (Supplementary Figure S1D). To separate these regions, the minimum between the two frequency regions was determined (Supplementary Figure S1B). Subsequently, the amplitudes above (to isolate the A- and B-wave) or below (to isolate the OPs) the minimum were set to zero. Inverse Fourier transforms were then performed on the two regions to obtain the ERG (Supplementary Figure S1C) and the isolated OPs (Supplementary Figure S1D), respectively. The curve obtained from the inverse Fourier transform of the low frequency fraction was used to measure the amplitude of the A- and B-wave (Supplementary Figure S1C). Implicit times were defined as the time between stimulus onset and the minimum of the A-wave or maximum of the B-wave, respectively.

Supplementary Figure S1.

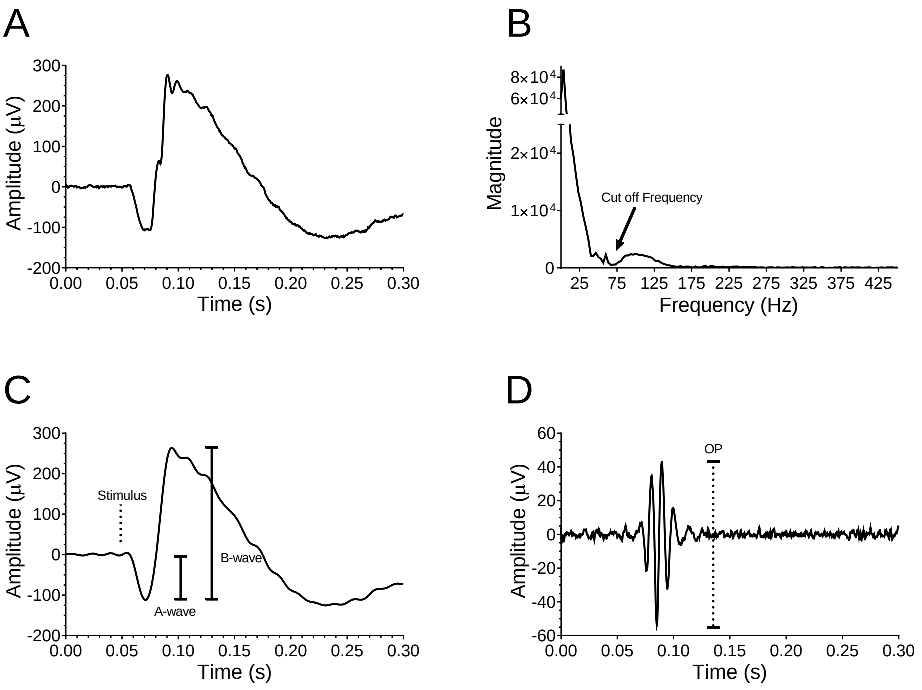

Supplementary Figure S1: Analysis of the ERG responses. (A) Original scotopic flash ERG response. (B) The amplitude plot obtained by FFT of the signal in (A) displays two distinct frequency regions. The low frequency region reflects the A- and B-wave and the high frequency region originates from the oscillatory potentials (OPs). (C) Reverse Fourier Transform of the low frequency region separates the a- and B-wave. The arrows indicate how A- and B-wave delays and amplitudes were measured. The dashed line indicates flash onset. (D) Reverse Fourier Transform of the high frequency region isolates the OPs.

Electroretinography (ERG): Derived Rod-Mediated B-wave (P2) Responses

To obtain the derived rod-mediated B-wave (P2) responses that are displayed in Supplementary Figure S4, a model of the leading edge of the A-wave (P3) was derived and removed from the ERG recording. To this end, we used a modified Lamb–Pugh model (equation 1 below) of the activation phase of transduction.

$$\boldsymbol{R(i\,,t) = R_{max}\,(1 - e^{(-i\left(\frac{t-t_d}{t_c}\right)^2}})\qquad \text{equation 1}$$

Where R_{max} (µV) is the maximal A-wave response, t_c (ms) is the characteristic time constant of transduction, t_d (ms) is the time delay, and I (cd s/m^2) is the stimulus intensity. R_{max} was set to the largest A-wave amplitude and t_c and t_d were allowed to vary.

$$\boldsymbol{R^*(i\,,t) = R(i\,,t) * P(t\,,t_p)}\qquad \text{equation 2}$$

Where P is a rectangular pulse function of duration t_p and total area equal to 1, and '*' is the convolution operation. The Ops were then removed with an FFT (Supplementary Figure 1).

SUPPLEMENTARY RESULTS

Both Ts65Dn and Dp16 mice display significantly increased inner retina thickness

Supplementary Figure S2.

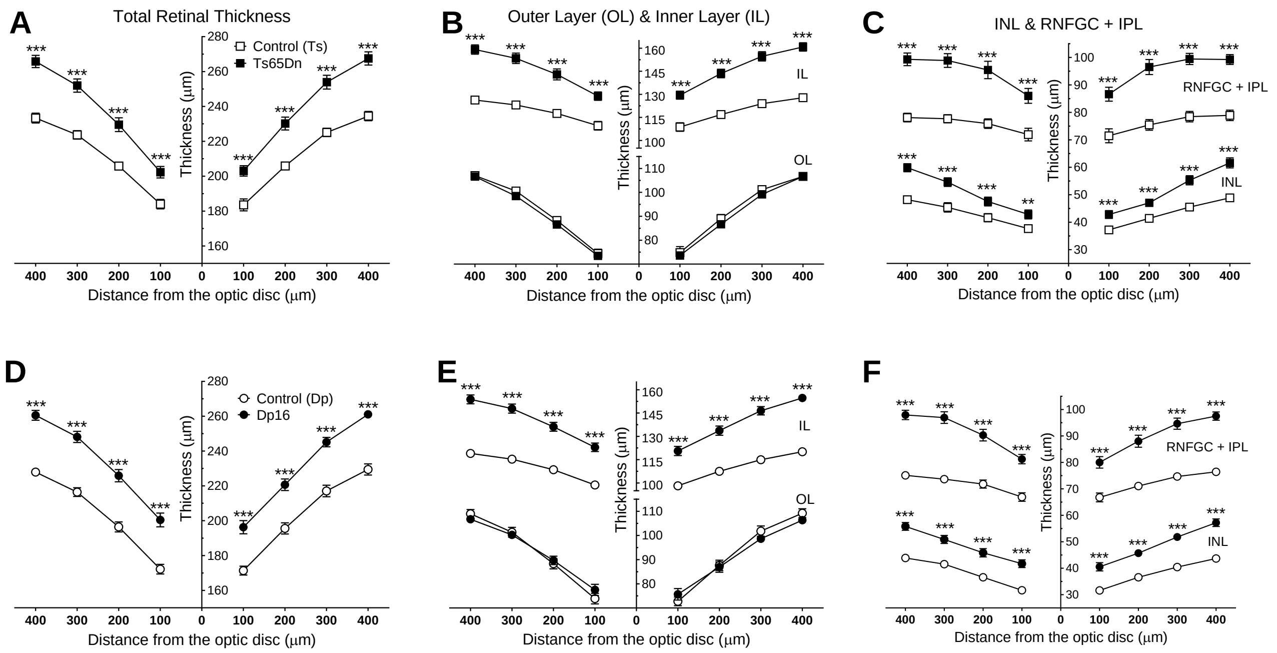

Supplementary Figure S2: Mean retinal thickness values obtained from 17-day old animals. Ts65Dn and Dp16 mice presented an increase in the total retinal thickness (A and D, respectively) compared with their respective age-matched control mice in all lateral distances considered. No genotype effect on the mean thickness of the outer retinal layer was observed for either chromosomally altered mice (lower portion of panels B and E). However, Ts65Dn and Dp16 mice showed a genotype-dependent increase in the thickness of the inner retina (upper portion of panels B and E, respectively), which includes increased thickness of the **three innermost retinal layers -** retinal nerve fiber ganglion cell (RNFGC) complex, inner plexiform layer (IPL), and inner nuclear layer (INL). Retinal layer thicknesses were plotted (mean ± SEM) with four lateral distances from the optic disc on both sides (100, 200, 300, and 400 µm). Statistical significance is expressed as ** and *** for $p<0.01$ and $p<0.001$, respectively.

Supplementary Figure S3.

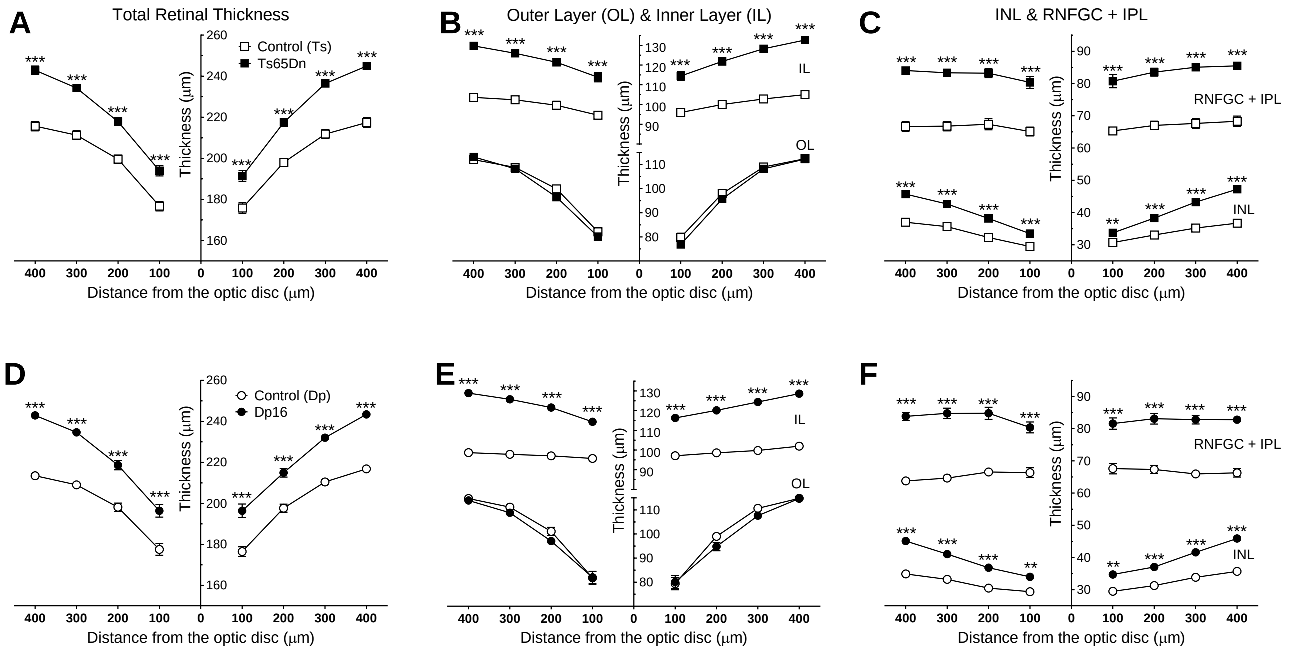

Supplementary Figure S3: Mean retinal thickness values obtained from adult animals. Ts65Dn and Dp16 mice presented an increase in the total retinal thickness (A and D, respectively) compared with their respective age-matched control mice in all lateral distances considered. No genotype effect on the mean thickness of the outer retinal layer was observed for either chromosomally altered mice (lower portion of panels B and E). However, Ts65Dn and Dp16 mice showed a genotype-dependent increase in the thickness of the inner retina (upper portion of panels B and E, respectively), which includes increased thickness of the **three innermost retinal layers -** retinal nerve fiber ganglion cell (RNFGC) complex, inner plexiform layer (IPL), and the inner nuclear layer (INL). Retinal layer thicknesses were plotted (mean ± SEM) with four lateral distances from the optic disc on both sides (100, 200, 300, and 400 µm). Statistical significance is expressed as ** and *** for $p<0.01$ and $p<0.001$, respectively.

Supplementary Table S1. Retinal thickness - OCT analysis.

Retinal thickness – OCT analysis				
Two-way Repeated Measures ANOVA				
	Effect	Degr. of (Freedom)	F	p
Total retina	Intercept	1	86481.522	< 0.0001
	Genotype	3	94.247	< 0.0001
	Age	2	29.597	< 0.0001
	Genotype*Age	6	1.359	0.2347
	Error	149		
	Distance	7	1604.932	< 0.0001
	Distance*Genotype	21	7.752	< 0.0001
	Distance*Age	14	19.188	< 0.0001
	Distance*Genotype*Age	42	0.698	0.9281
	Error	1043		
Outer retina	Intercept	1	56411.664	< 0.0001
	Genotype	3	1.079	0.3598
	Age	2	33.153	< 0.0001
	Genotype*Age	6	0.098	0.9964
	Error	149		
	Distance	7	1269.937	< 0.0001
	Distance*Genotype	21	1.352	0.1330
	Distance*Age	14	5.132	< 0.0001
	Distance*Genotype*Age	42	0.901	0.6531
	Error	1043		
Inner retina	Intercept	1	45926.951	< 0.0001
	Genotype	3	185.255	< 0.0001
	Age	2	117.321	< 0.0001
	Genotype*Age	6	1.934	0.0790
	Error	149		
	Distance	7	306.959	< 0.0001
	Distance*Genotype	21	10.440	< 0.0001
	Distance*Age	14	27.532	< 0.0001
	Distance*Genotype*Age	42	0.994	0.4829
	Error	1043		
Inner nuclear layer	Intercept	1	28436.830	< 0.0001
	Genotype	3	107.771	< 0.0001
	Age	2	164.992	< 0.0001
	Genotype*Age	6	3.147	0.0062
	Error	149		
	Distance	7	399.680	< 0.0001
	Distance*Genotype	21	9.756	< 0.0001
	Distance*Age	14	12.129	< 0.0001
	Distance*Genotype*Age	42	0.528	0.9945
	Error	1043		
Retinal nerve fiber ganglion cell complex	Intercept	1	12890.123	< 0.0001
	Genotype	3	44.012	< 0.0001
	Age	2	54.092	< 0.0001
	Genotype*Age	6	3.931	0.0011
	Error	149		

	Distance	7	31.873	< 0.0001
	Distance*Genotype	21	0.602	0.9193
	Distance*Age	14	4.061	< 0.0001
	Distance*Genotype*Age	42	1.551	0.0147
	Error	1043		
Inner plexiform layer	Intercept	1	15353.271	< 0.0001
	Genotype	3	71.442	< 0.0001
	Age	2	9.936	< 0.0001
	Genotype*Age	6	1.824	0.0980
	Error	149		
	Distance	7	107.916	< 0.0001
	Distance*Genotype	21	4.668	< 0.0001
	Distance*Age	14	10.955	< 0.0001
	Distance*Genotype*Age	42	1.039	0.4039
	Error	1043		
Retinal nerve fiber ganglion cell complex and inner plexiform layer	Intercept	1	30987.021	< 0.0001
	Genotype	3	129.575	< 0.0001
	Age	2	43.726	< 0.0001
	Genotype*Age	6	1.570	0.1598
	Error	149		
	Distance	7	54.365	< 0.0001
	Distance*Genotype	21	4.200	< 0.0001
	Distance*Age	14	15.588	< 0.0001
	Distance*Genotype*Age	42	1.329	0.0802
	Error	1043		

Supplementary Table S2. Fisher's least significant difference (LSD) post hoc analysis (Retinal thickness - OCT analysis)

Retinal thickness – OCT analysis							
Fisher's least significant difference (LSD) post hoc analysis							
Genotype	Retinal layer	Age	Lateral distances (p valu				
			Left side				
			100	200	300	400	100
Control (Ts) *vs* Ts65Dn	Total retina	17	< 0.0001	< 0.0001	< 0.0001	< 0.0001	< 0.0001
		35	0.0001	< 0.0001	< 0.0001	< 0.0001	< 0.0001
		Adults	< 0.0001	< 0.0001	< 0.0001	< 0.0001	< 0.0001
	Inner retina	17	< 0.0001	< 0.0001	< 0.0001	< 0.0001	< 0.0001
		35	< 0.0001	< 0.0001	< 0.0001	< 0.0001	< 0.0001
		Adults	< 0.0001	< 0.0001	< 0.0001	< 0.0001	< 0.0001
	Inner nuclear layer	17	0.0017	0.0004	< 0.0001	< 0.0001	0.0007
		35	0.0120	0.0007	< 0.0001	< 0.0001	0.0004
		Adults	0.0006	< 0.0001	< 0.0001	< 0.0001	0.0094
	Retinal nerve fiber ganglion cell complex	17	< 0.0001	< 0.0001	< 0.0001	< 0.0001	< 0.0001
		35	0.2438	0.0349	0.0108	0.0048	0.1943
		Adults	0.0043	0.0120	0.0268	0.0069	0.0002
	Inner plexiform layer	17	0.0448	0.0008	< 0.0001	< 0.0001	0.0304
		35	< 0.0001	< 0.0001	< 0.0001	< 0.0001	< 0.0001
		Adults	< 0.0001	< 0.0001	< 0.0001	< 0.0001	< 0.0001
	Retinal nerve fiber ganglion cell complex and inner plexiform layer	17	< 0.0001	< 0.0001	< 0.0001	< 0.0001	< 0.0001
		35	< 0.0001	< 0.0001	< 0.0001	< 0.0001	< 0.0001
		Adults	< 0.0001	< 0.0001	< 0.0001	< 0.0001	< 0.0001
Control (Dp) *vs* Dp16	Total retina	17	< 0.0001	< 0.0001	< 0.0001	< 0.0001	< 0.0001
		35	0.0016	0.0008	0.0001	< 0.0001	0.0001
		Adults	< 0.0001	< 0.0001	< 0.0001	< 0.0001	< 0.0001
	Inner retina	17	< 0.0001	< 0.0001	< 0.0001	< 0.0001	< 0.0001
		35	< 0.0001	< 0.0001	< 0.0001	< 0.0001	< 0.0001
		Adults	< 0.0001	< 0.0001	< 0.0001	< 0.0001	< 0.0001
	Inner nuclear layer	17	< 0.0001	< 0.0001	< 0.0001	< 0.0001	< 0.0001

	35	< 0.0001	< 0.0001	< 0.0001	< 0.0001	< 0.0001
	Adults	0.0030	< 0.0001	< 0.0001	< 0.0001	0.0021
Retinal nerve fiber ganglion cell complex	17	< 0.0001	< 0.0001	< 0.0001	< 0.0001	< 0.0001
	35	0.0020	0.0084	0.0410	0.0026	< 0.0001
	Adults	0.0069	0.0003	0.0046	0.0182	0.0002
Inner plexiform layer	17	0.0056	0.0006	< 0.0001	< 0.0001	0.0125
	35	0.0286	0.0106	0.0006	0.0001	0.0340
	Adults	< 0.0001	< 0.0001	< 0.0001	< 0.0001	< 0.0001
Retinal nerve fiber ganglion cell complex and inner plexiform layer	17	< 0.0001	< 0.0001	< 0.0001	< 0.0001	< 0.0001
	35	0.0002	0.0001	< 0.0001	< 0.0001	< 0.0001
	Adults	< 0.0001	< 0.0001	< 0.0001	< 0.0001	< 0.0001

Ts65Dn and Dp16 mice present mild, but significant alterations in retinal vasculature

Supplementary Table S3. Retinal vasculature parameters - SIVA analysis

Retinal vasculature parameters - SIVA analysis				
Two-way Repeated Measures Analysis of Variance				
Parameter	Effect	Degr. of (Freedom)	F	p
Number of vessels	Intercept	1	5196.806	< 0.0001
	Genotype	3	2.207	0.1035
	Error	37		
	Age	1	1.096	0.3019
	Age*Genotype	3	1.031	0.3900
	Error	37		
Vasculature caliber	Intercept	1	17518.235	< 0.0001
	Genotype	3	20.644	< 0.0001
	Error	37		
	Age	1	5.262	0.0276
	Age*Genotype	3	1.629	0.1992
	Error	37		
Vasculature fractal dimension	Intercept	1	68151.498	< 0.0001
	Genotype	3	2.251	0.0986
	Error	37		
	Age	1	0.385	0.5388
	Age*Genotype	3	0.905	0.4482
	Error	37		
Vascular curvature tortuosity	Intercept	1	2553.056	< 0.0001
	Genotype	3	1.355	0.2717
	Error	37		
	Age	1	0.201	0.6566
	Age*Genotype	3	1.572	0.2126
	Error	37		

Supplementary Table S4. Fisher's least significant difference (LSD) post hoc analysis (Vasculature caliber - SIVA analysis)

Vasculature caliber - SIVA analysis		
Fisher's least significant difference (LSD) post hoc analysis		
Genotype	Age	
	35	Adults
Control (Ts) *vs* Ts65Dn	0.0006	0.0017
Control (Dp) *vs* Dp16	0.0464	0.0001
Ts65Dn *vs* Ts65Dn		0.0321
Dp16 *vs* Dp16		0.4931
Ts65Dn *vs* Dp16	0.6132	0.0009

Supplementary Table S5. Early postnatal development of the primary plexus analysis (Angiotool, Image J, and IgorPro analysis)

Early postnatal development of the primary plexus analysis				
Two-way Repeated Measures Analysis of Variance				
Parameter	Effect	Degr. of (Freedom)	F	p
Vascularized retinal area	Intercept	1	3770.131	< 0.0001
	Genotype	3	1.144	0.3452
	Error	34		
	Age	3	447.625	< 0.0001
	Age*Genotype	9	0.499	0.8725
	Error	102		
Avascularized retinal area	Intercept	1	4568.750	< 0.0001
	Genotype	3	2.999	0.0441
	Error	34		
	Age	3	303.192	< 0.0001
	Age*Genotype	9	3.307	0.0014
	Error	102		
Total retinal area	Intercept	1	8530.166	< 0.0001
	Genotype	3	0.630	0.6004
	Error	34		
	Age	3	151.765	< 0.0001
	Age*Genotype	9	1.970	0.0504
	Error	102		
Lacunarity	Intercept	1	3318.435	< 0.0001
	Genotype	3	1.934	0.1439
	Error	32		
	Age	3	70.014	< 0.0001
	Age*Genotype	9	1.554	0.1403
	Error	96		
Total vessel length	Intercept	1	2783.945	< 0.0001
	Genotype	3	0.720	0.5476
	Error	32		
	Age	3	380.867	< 0.0001
	Age*Genotype	9	0.394	0.9355
	Error	96		
Vessel caliber	Intercept	1	23423.984	< 0.0001
	Vessel	1	1569.337	< 0.0001
	Genotype	3	4.712	0.0046
	Vessel*Genotype	3	2.822	0.0445
	Error	75		
	Age	1	122.214	< 0.0001
	Age*Vessel	1	53.195	< 0.0001
	Age*Genotype	3	0.156	0.9258

	Age*Vessel*Genotype	3	0.551	0.6493
	Error	75		
Number of vessels	Intercept	1	8420.152	< 0.0001
	Genotype	3	10.824	< 0.0001
	Vessel	1	5.048	0.0276
	Genotype*Vessel	3	0.053	0.9840
	Error	75		
	Age	1	7.821	0.0066
	Age*Genotype	3	2.091	0.1085
	Age*Vessel	1	1.348	0.2494
	Age*Genotype*Vessel	3	0.249	0.8621
	Error	75		

Supplementary Table S6. Fisher's least significant difference (LSD) post hoc analysis (Avascularized retinal area - Angiotool analysis)

Avascularized retinal area - Angiotool analysis				
Fisher's least significant difference (LSD) post hoc analysis				
Genotype	Age			
	1	3	6	9
Control (Ts) *vs* Ts65Dn	0.6016	0.9067	0.3350	0.4577
Control (Dp) *vs* Dp16	0.4888	0.1243	0.3633	0.7452
Ts65Dn *vs* Dp16	0.0007	0.7181	0.4701	0.4694

Supplementary Table S7. Fisher's least significant difference (LSD) post hoc analysis (Vessel caliber - IgorPro analysis)

Vessel caliber - IgorPro analysis			
Fisher's least significant difference (LSD) post hoc analysis			
Genotype	Vessel type	Age	
		6	9
Control (Ts) *vs* Ts65Dn	Arteriole	0.3856	0.7995
	Venule	0.0008	0.0045
Control (Dp) *vs* Dp16	Arteriole	0.7640	0.8242
	Venule	0.6899	0.2969

Supplementary Table S8. Fisher's least significant difference (LSD) post hoc analysis (Number of vessels - IgorPro analysis)

Number of vessels - IgorPro analysis			
Fisher's least significant difference (LSD) post hoc analysis			
Genotype	Vessel type	Age	
		6	9
Control (Ts) *vs* Ts65Dn	Arteriole	0.1283	0.0209
	Venule	0.0664	0.1524
Control (Dp) *vs* Dp16	Arteriole	0.0019	0.6711
	Venule	0.0083	0.5442

Ts65Dn and Dp16 mice display mildly altered scotopic ERGs

Supplementary Table S9. Urethane: Ts65Dn and Dp16 (Two-way RM ANOVA)

Urethane: Ts65Dnand Dp16				
Two-way Repeated Measures Analysis of Variance				
Waveform	Effect	Degr. of (Freedom)	F	p
A-Wave	Intercept	1	422.484	< 0.0001
	Genotype	3	3.795	0.0167
	Error	44		
	Stimulus	7	251.917	< 0.0001
	Stimulus*Genotype	21	2.847	< 0.0001
	Error	308		
B-Wave	Intercept	1	296.581	< 0.0001
	Genotype	3	0.394	0.7575
	Error	44		
	Stimulus	16	261.851	< 0.0001
	Stimulus*Genotype	48	0.356	0.9999
	Error	704		
OPs	Intercept	1	490.577	< 0.0001
	Genotype	3	3.853	0.0156
	Error	44		
	Stimulus	14	203.534	< 0.0001
	Stimulus*Genotype	42	2.715	< 0.0001
	Error	616		

Supplementary Table S10. Urethane: Ts65Dn and Dp16 A-Wave (Fisher's LSD post hoc analysis)

Urethane: Ts65Dn vs. Dp16 A-Wave		
Post-hoc (Fisher's least significant difference)		
Genotype	Stimulus Intensity (log cd·s/m^2)	p
Ts65Dn vs. Control (Ts)	-1.1	0.6027
	-0.8	0.6458
	-0.5	0.2405
	-0.2	0.1641
	0.1	0.1912
	0.4	0.0428
	0.7	0.0084
	1.0	0.0002
Dp16 vs. Control (Dp)	-1.1	0.7298
	-0.8	0.5887
	-0.5	0.4844
	-0.2	0.3865
	0.1	0.0451

	0.4	0.0948
	0.7	0.0049
	1.0	< 0.0001
Ts65Dn vs. Dp16	-1.1	0.7263
	-0.8	0.7274
	-0.5	0.4010
	-0.2	0.2243
	0.1	0.6310
	0.4	0.0398
	0.7	0.3606
	1.0	0.2516
Control (Ts) vs. Control (Dp)	-1.1	0.8612
	-0.8	0.6674
	-0.5	0.7161
	-0.2	0.4913
	0.1	0.2351
	0.4	0.0890
	0.7	0.2695
	1.0	0.1607

Supplementary Table S11. Urethane: Ts65Dn vs. Dp16 OPs (Fisher's LSD post hoc analysis)

Urethane: Ts65Dn vs. Dp16 OPs		
Post-hoc (Fisher's least significant difference)		
Genotype	Stimulus Intensity (log cd·s/m^2)	p
Ts65Dn vs. Control (Ts)	-3.2	0.7663
	-2.9	0.9964
	-2.6	0.7387
	-2.3	0.5675
	-2.0	0.7623
	-1.7	0.4752
	-1.4	0.3756
	-1.1	0.0639
	-0.8	0.0149
	-0.5	0.0072
	-0.2	0.0011
	0.1	0.0024
	0.4	< 0.0001
	0.7	< 0.0001
	1.0	< 0.0001
Dp16 vs. Control (Dp)	-3.2	0.7010
	-2.9	0.6005
	-2.6	0.4121
	-2.3	0.2664
	-2.0	0.1080
	-1.7	0.3454
	-1.4	0.1115
	-1.1	0.3865
	-0.8	0.3537

	-0.5	0.3182
	-0.2	0.1481
	0.1	0.1123
	0.4	0.0042
	0.7	0.0108
	1.0	0.0088
Ts65Dn vs. Dp16	-3.2	0.7857
	-2.9	0.8826
	-2.6	0.9583
	-2.3	0.9599
	-2.0	0.4398
	-1.7	0.4786
	-1.4	0.7720
	-1.1	0.3975
	-0.8	0.2676
	-0.5	0.3329
	-0.2	0.4400
	0.1	0.8128
	0.4	0.9882
	0.7	0.9222
	1.0	0.4402
Control (Ts) vs. Control (Dp)	-3.2	0.7198
	-2.9	0.4993
	-2.6	0.5896
	-2.3	0.5542
	-2.0	0.5904
	-1.7	0.3482
	-1.4	0.3178
	-1.1	0.8812
	-0.8	0.6745
	-0.5	0.4536
	-0.2	0.2718
	0.1	0.2112
	0.4	0.2670
	0.7	0.0458
	1.0	0.0714

Supplementary Table S12. Urethane: Ts65Dn and Dp16 (One-way ANOVA)

Urethane: Ts65Dn and Dp16				
One-way Analysis of Variance				
Variables	Effect	Degr. of (Freedom)	F	p
R_{max}	Intercept	1	391.472	< 0.0001
	Genotype	3	3.132	0.0359
	Error	44		
t_c	Intercept	1	1412.906	< 0.0001
	Genotype	3	2.452	0.0759
	Error	44		
t_d	Intercept	1	3097.943	< 0.0001
	Genotype	3	1.259	0.3000
	Error	44		

Supplementary Table S13. Urethane: Ts65Dn and Dp16 (Fisher's LSD post hoc analysis)

Urethane: Ts65Dn vs. Dp16 R_{max}				
Fisher's least significant difference (LSD) post hoc analysis				
Genotype	Genoytype			
	Control (Ts)	Ts656Dn	Control (Dp)	Dp16
Control (Ts)		0.0426	0.1416	0.5601
Ts656Dn	0.0426		0.5574	0.0104
Control (Dp)	0.1416	0.5574		0.0430
Dp16	0.5601	0.0104	0.0430	

Supplementary Table S14. Naka-Rushton Analysis B-wave

Naka-Rushton Analysis B-wave				
Two-way Analysis of Variance				
Genotype/Anesthetic	Variable	Degr. of (Freedom)	F	p
Urethane Ts65Dn	V_{max}	1	0.221	0.6382
	K	1	0.001	0.9726
	Slope	1	0.031	0.8594
	Error	402		
Urethane Dp16	V_{max}	1	0.0003	0.9861
	K	1	0.082	0.7730
	Slope	1	0.175	0.6760
	Error	402		

Scotopic ERG properties in control (wild-type) mice are dependent on the type of anesthetic agent used in the experiment

Supplementary Table S15. Ts65Dn: Urethane vs. Ketamine vs. TBE (Two-way RM ANOVA)

Ts65Dn: Urethane vs. Ketamine vs. TBE				
Two-way Repeated Measures Analysis of Variance				
Waveform	Effect	Degr. of (Freedom)	F	p
A-Wave	Intercept	1	1074.944	< 0.0001
	Genotype	1	6.609	0.0124
	Anesthetic	2	2.751	0.0711
	Genotype*Anesthetic	2	1.211	0.3045
	Error	67		
	Stimulus	7	413.031	< 0.0001
	Stimulus*Genotype	7	5.638	< 0.0001
	Stimulus*Anesthetic	14	1.732	0.0465
	Stimulus*Genotype*Anesthetic	14	0.710	0.7654
	Error	469		
B-Wave	Intercept	1	695.359	< 0.0001
	Genotype	1	0.036	0.8502
	Anesthetic	2	2.896	0.0622
	Genotype*Anesthetic	2	0.361	0.6981
	Error	67		
	Stimulus	16	540.270	< 0.0001
	Stimulus*Genotype	16	1.645	0.0517
	Stimulus*Anesthetic	32	5.207	< 0.0001
	Stimulus*Genotype*Anesthetic	32	0.953	0.5430
	Error	1072		
OPs	Intercept	1	756.578	< 0.0001
	Genotype	1	23.499	< 0.0001
	Anesthetic	2	15.881	< 0.0001
	Genotype*Anesthetic	2	1.467	0.2379
	Error	67		
	Stimulus	14	310.441	< 0.0001
	Stimulus*Genotype	14	14.695	< 0.0001
	Stimulus*Anesthetic	28	9.828	< 0.0001
	Stimulus*Genotype*Anesthetic	28	1.895	0.0035
	Error	938		

Supplementary Table S16. Ts65Dn: Urethane vs. Ketamine vs. TBE OPs (Fisher's LSD post hoc analysis)

Ts65Dn: Urethane vs. Ketamine vs. TBE OPs		
Post-hoc (Fisher's least significant difference)		
Genotype/Anesthetic	Stimulus Intensity (log cd·s/m^2)	p
Ts65Dn vs. Control (Ts) Urethane	-3.2	0.8130
	-2.9	0.9972
	-2.6	0.7905
	-2.3	0.6489
	-2.0	0.8097
	-1.7	0.5696
	-1.4	0.4803
	-1.1	0.1389
	-0.8	0.0512
	-0.5	0.0313
	-0.2	0.0086
	0.1	0.0147
	0.4	0.0015
	0.7	0.0004
	1.0	< 0.0001
Ts65Dn vs. Control (Ts) Ketamine	-3.2	0.7573
	-2.9	0.6170
	-2.6	0.4105
	-2.3	0.3598
	-2.0	0.3702
	-1.7	0.0968
	-1.4	0.0169
	-1.1	0.0323
	-0.8	0.0017
	-0.5	< 0.0001
	-0.2	< 0.0001
	0.1	< 0.0001
	0.4	< 0.0001
	0.7	< 0.0001
	1.0	0.0008
Ts65Dn vs. Control (Ts) TBE	-3.2	0.8798
	-2.9	0.7226
	-2.6	0.4547
	-2.3	0.1431
	-2.0	0.0648
	-1.7	0.4229
	-1.4	0.9321
	-1.1	0.1948
	-0.8	0.1589
	-0.5	0.0423
	-0.2	0.0358
	0.1	0.0035
	0.4	0.0003

	0.7	0.0224
	1.0	0.0292
	-3.2	0.9672
	-2.9	0.7175
	-2.6	0.4742
	-2.3	0.3990
	-2.0	0.2818
	-1.7	0.7831
	-1.4	0.7171
Ts65Dn Urethane vs. Ts65Dn Ketamine	-1.1	0.8734
	-0.8	0.9611
	-0.5	0.3959
	-0.2	0.0880
	0.1	0.0002
	0.4	< 0.0001
	0.7	< 0.0001
	1.0	< 0.0001
	-3.2	0.7974
	-2.9	0.6882
	-2.6	0.1359
	-2.3	0.0271
	-2.0	0.0012
	-1.7	0.0033
	-1.4	0.0032
Ts65Dn Urethane vs. Ts65Dn TBE	-1.1	0.0024
	-0.8	0.0057
	-0.5	0.0003
	-0.2	0.0006
	0.1	0.0001
	0.4	< 0.0001
	0.7	0.0003
	1.0	< 0.0001
	-3.2	0.8299
	-2.9	0.9742
	-2.6	0.4448
	-2.3	0.1751
	-2.0	0.0306
	-1.7	0.0076
	-1.4	0.0096
Ts65Dn Ketamine vs. Ts65Dn TBE	-1.1	0.0040
	-0.8	0.0066
	-0.5	0.0052
	-0.2	0.0859
	0.1	0.9926
	0.4	0.2709
	0.7	0.0244
	1.0	0.6651
	-3.2	0.9749
	-2.9	0.8871
Control (Ts) Urethane vs. Control (Ts) Ketamine	-2.6	0.8745
	-2.3	0.7019
	-2.0	0.6733
	-1.7	0.4116

	-1.4	0.1822
	-1.1	0.6106
	-0.8	0.2473
	-0.5	0.3016
	-0.2	0.3925
	0.1	0.5370
	0.4	0.3245
	0.7	0.0020
	1.0	< 0.0001
Control (Ts) Urethane vs. Control (Ts) TBE	-3.2	0.5246
	-2.9	0.4607
	-2.6	0.3189
	-2.3	0.2339
	-2.0	0.1037
	-1.7	0.0075
	-1.4	0.0005
	-1.1	0.0015
	-0.8	0.0011
	-0.5	0.0002
	-0.2	< 0.0001
	0.1	0.0009
	0.4	0.0003
	0.7	< 0.0001
	1.0	< 0.0001
Control (Ts) Ketamine vs. Control (Ts) TBE	-3.2	0.5043
	-2.9	0.3792
	-2.6	0.4013
	-2.3	0.4185
	-2.0	0.2268
	-1.7	0.0005
	-1.4	< 0.0001
	-1.1	0.0002
	-0.8	< 0.0001
	-0.5	< 0.0001
	-0.2	< 0.0001
	0.1	< 0.0001
	0.4	0.0081
	0.7	0.0661
	1.0	0.4052

Supplementary Table S17. Ts65Dn: Urethane vs. Ketamine vs. TBE (Two-way ANOVA)

Ts65Dn: Urethane vs. Ketamine vs. TBE				
Two-way Analysis of Variance				
Waveform	Effect	Degr. of (Freedom)	F	p
R_{max}	Intercept	1	988.643	< 0.0001
	Genotype	1	9.555	0.0029
	Anesthetic	2	0.129	0.8793
	Genotype*Anesthetic	2	0.970	0.3842
	Error	67		
t_c	Intercept	1	1530.246	< 0.0001
	Genotype	1	1.000	0.3210
	Anesthetic	2	11.160	< 0.0001
	Genotype*Anesthetic	2	0.851	0.4315
	Error	67		
t_d	Intercept	1	6319.281	< 0.0001
	Genotype	1	1.840	0.1795
	Anesthetic	2	2.153	0.1241
	Genotype*Anesthetic	2	1.277	0.2856
	Error	67		

Supplementary Table S18. Ts65Dn: Urethane vs. Ketamine vs. TBE (Fisher's LSD post hoc analysis)

Ts65Dn: Urethane vs. Ketamine vs. TBE R_{max}						
Fisher's least significant difference (LSD) post hoc analysis						
Genotype/Anesthetic	Genotype/Anesthetic					
	Ct Ure	Ct Ket	Ct TBE	Ts Ure	Ts Ket	Ts TBE
Control Urethane		0.3088	0.2169	0.0054	0.0161	0.0250
Control Ketamine	0.3088		0.8257	0.0689	0.1536	0.2168
Control TBE	0.2169	0.8257		0.1082	0.2259	0.3107
Ts65Dn Urethane	0.0054	0.0689	0.1082		0.6861	0.5251
Ts65Dn Ketamine	0.0161	0.1536	0.2259	0.6861		0.8228
Ts65Dn TBE	0.0250	0.2168	0.3107	0.5251	0.8228	

Supplementary Table S19. Naka-Rushton Analysis B-wave - Two-way Analysis of Variance

Naka-Rushton Analysis B-wave				
Genotype/Anesthetic	Variable	Degr. of (Freedom)	F	p
Ketamine Ts65Dn	V_{max}	1	1.505	0.2207
	K	1	0.032	0.8565
	Slope	1	3.545	0.0604
	Error	402		
TBE Ts65Dn	V_{max}	1	2.939	0.0872
	K	1	0.230	0.2300
	Slope	1	0.540	0.5399
	Error	419		

Photopic ERG properties are not altered in Ts65Dn and Dp16 mice

Supplementary Table S20. Urethane: Photopic ERG Ts65Dn (One-way ANOVA)

Urethane: Photopic ERG (One-way ANOVA): Ts65Dn				
One-way Repeated Measures Analysis of Variance				
Waveform	Effect	Degr. of (Freedom)	F	p
Photopic ERG B-Wave	Intercept	1	268.186	< 0.0001
	Genotype	1	0.646	0.4301
	Error	22		
	Stimulus	8	234.134	< 0.0001
	Stimulus*Genotype	8	0.4973	0.8571
	Error	176		

Supplementary Table S21. Urethane: Photopic ERG Dp16 (One-way ANOVA)

Urethane: Photopic ERG (One-way ANOVA): Dp16				
One-way Repeated Measures Analysis of Variance				
Waveform	Effect	Degr. of (Freedom)	F	p
Photopic ERG B-Wave	Intercept	1	265.358	< 0.0001
	Genotype	3	0.030	0.8650
	Error	44		
	Stimulus	7	155.950	< 0.0001
	Stimulus*Genotype	21	0.6164	0.7634
	Error	308		

Supplementary Table S22. Photopic ERG Naka-Rushton Analysis - B-wave

Naka-Rushton Analysis B-wave				
Genotype/Anesthetic	Variable	Degr. of (Freedom)	F	p
Photopic ERG Ts65Dn	V_{max}	1	0.119	0.7300
	K	1	0.603	0.4384
	Slope	1	0.792	0.7923
	Error	210		
Photopic ERG Dp16	V_{max}	1	0.563	0.4540
	K	1	0.357	0.5510
	Slope	1	0.059	0.8084
	Error	210		

Derived Rod-Mediated B-wave (P2) Responses

Ts65Dn and Dp16 mice showed significant decreased maximum rod-mediated responses (R_{max}) when compared with their respective euploid control mice (see Supplementary Tables S12 and S13). For the rod-mediated B-wave, analysis revealed a significant stimulus-intensity dependence ($p < 0.0001$), however, no significant genotype dependence ($p = 0.7738$) or significant interaction between stimulus intensity and genotype ($p = 0.5447$) (Supplementary Table S23) was found (Supplementary Figures 4E and 4F). A two-way ANOVA did not show a significant difference in maximum rod-mediated response (R_{max}) between Ts65Dn and Control (Ts) mice for either anesthetic agent (ketamine and TBE) (Figures 6 C, 7 C, and 7 H) (see Supplementary Table S17 and S18 for statistics). Analysis of the rod-mediated B-wave revealed a significant stimulus-intensity dependence ($p < 0.0001$), but no significant dependence on genotype ($p = 0.8393$) or anesthetic agent ($p = 0.0760$) (Supplementary Table S24). In addition, we did not find a significant difference between anesthetic agent and genotype ($p = 0.0458$) but there was a significant difference between stimulus intensity and genotype ($p < 0.0001$) (Supplementary Table S24 and Supplementary Figures 4E, 4G, and 4H).

Supplementary Figure S4.

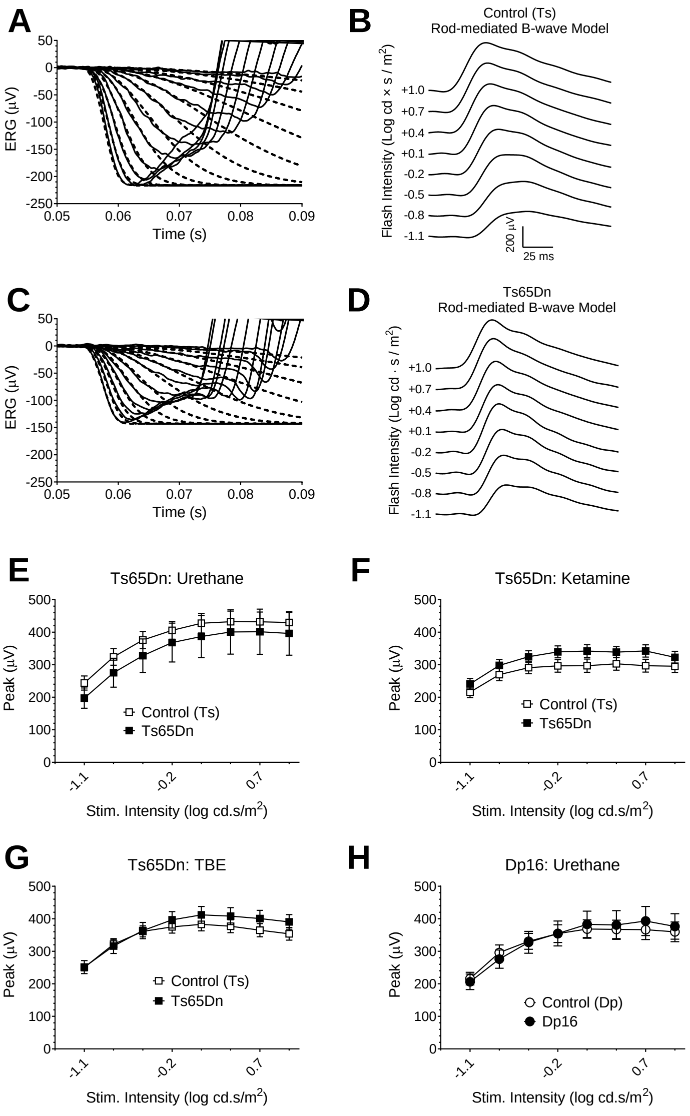

Supplementary Figure S4: Analysis of rod-mediated function in dark-adapted Ts65Dn, Dp16 mice, and their euploid control mice. Representative traces (solid lines) from (A) control (Ts) and fitted with a model (dashed lines). (B) Rod ERG traces after the A-wave function and OPs were removed. Representative traces (solid lines) from (C) Ts65Dn and fitted with a model (dashed lines). (D) Rod ERG traces after the A-wave function and OPs were removed. No difference in the amplitude of the rod-mediated B-Wave was detected in Ts65Dn when compared with control (Ts) mice for (E) urethane, (F) ketamine, or (G) TBE. (H) Dp16 did not show a difference in rod-mediated amplitude when compared with their euploid control mice. Mean ERG ± SEM peak amplitudes for euploid control (Ts) (n = 12), Ts65Dn (n = 12) under urethane anesthesia, control (Ts) (n = 12), Ts65Dn (n = 12) under ketamine anesthesia, control (Ts) (n = 12), Ts65Dn (n = 13) under TBE anesthesia, and control (Dp) (n = 12) and Dp16 (n = 12) mice under urethane anesthesia.

Supplementary Table S23. Urethane: Rod-mediated B-wave (One-way ANOVA)

Rod-mediated B-wave Urethane: Ts65Dn and Dp16				
One-way Repeated Measures Analysis of Variance				
Waveform	Effect	Degr. of (Freedom)	F	p
B-Wave	Intercept	1	329.350	< 0.0001
	Genotype	3	0.372	0.7738
	Error	44		
	Stimulus	7	150.543	< 0.0001
	Stimulus*Genotype	21	0.9358	0.5447
	Error	308		

Supplementary Table S24. Ts65Dn: Rod-mediated B-wave - Urethane vs. Ketamine vs. TBE

Ts65Dn: Rod-mediated B-wave Urethane vs. Ketamine vs. TBE				
Two-way Repeated Measures Analysis of Variance				
Waveform	Effect	Degr. of (Freedom)	F	p
B-wave	Intercept	1	774.981	< 0.0001
	Genotype	1	0.041	0.8393
	Anesthetic	2	2.679	0.0760
	Genotype*Anesthetic	2	0.857	0.429
	Error	67		
	Stimulus	7	219.857	< 0.0001
	Stimulus*Genotype	7	2.066	0.0458
	Stimulus*Anesthetic	14	13.096	< 0.0001
	Stimulus*Genotype*Anesthetic	14	0.464	0.9513
	Error	469		

MRI of the eye did not reveal any gross genotype-dependent morphometric alterations in Ts65Dn and Dp16 mice

Supplementary Figure S5.

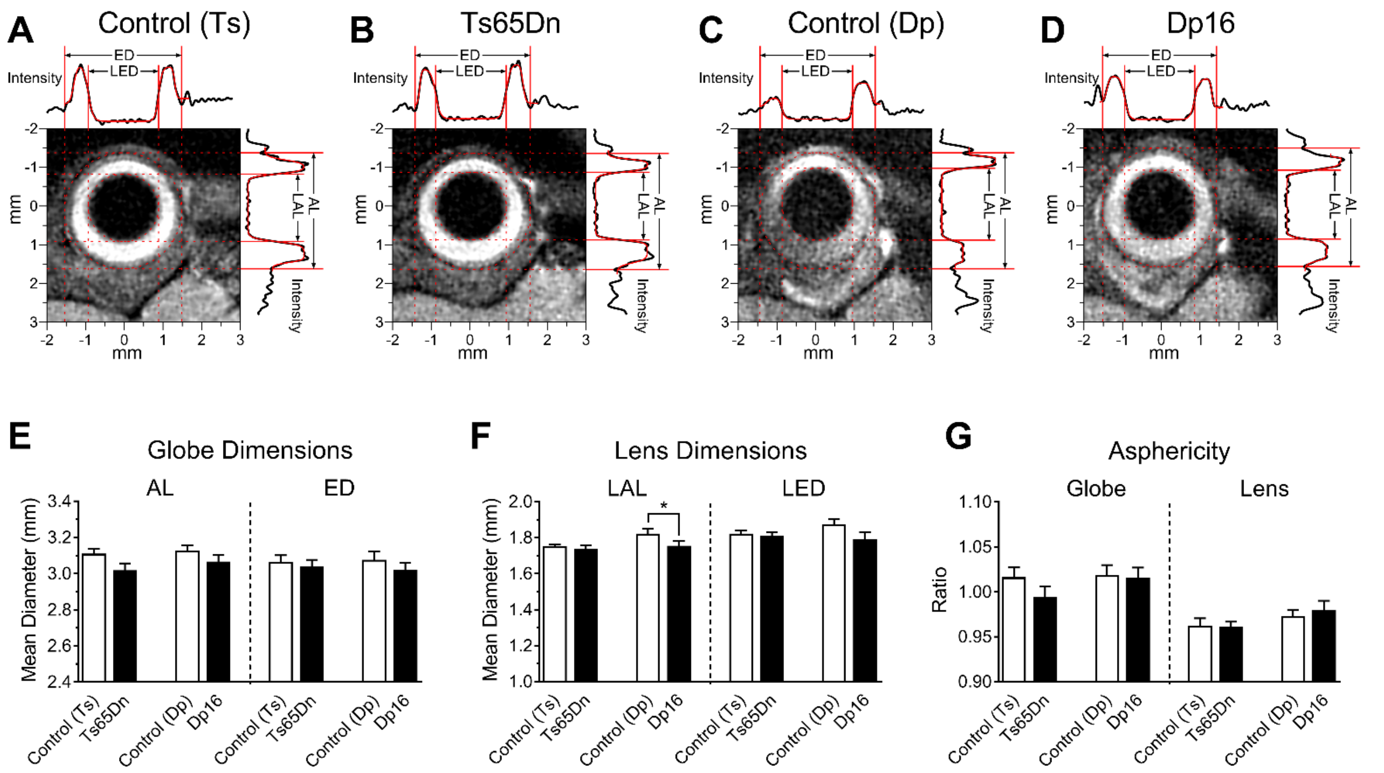

Supplementary Figure S5:

No significant differences in axial length (AL), equatorial distance (ED), or asphericity were observed between either Ts65Dn or Dp16 mice and their respective euploid controls. The measures of AL and ED were taken as the distance between the hypo-intense edge just outside the anterior and vitreous chambers. The lens axial length (LAL) and lens equatorial distance (LED) were taken as the distance between each inner pair of sigmoids at the mid-intensity (50%) point along the continuous curves. Asphericity of the whole eye and lens were calculated as the axial/equatorial dimension ratios. Example MRI scans and dimensional fits from two littermate pairs: Control (Ts), Ts65Dn, Control (Dp), and Dp16 (panels A, B, C, and D respectively). MRI protocol: view=prone; repetition time=4000ms; turbo factor=4x; effective echo interval=16ms; in-plane resolution=80x80μm; slice thickness=640μm; repetitions=2. A small, but significant difference in the LAL was found between Dp16 and Control (Dp) mice (Tables S21 and S22), which would need further corroboration, given the limitations in axial resolution of the MRI procedure used in the present study. Although there is a minor correlation between eye size and distance between the eyes, the slope is considerably less than 1/3 of what would be expected if there were proportionate scaling. There are significant differences between the Control (Ts) and Ts65Dn groups for eye distance ($p<0.05$) and weight ($p<0.001$), but no significant differences between the Control (Dp) and Dp16 groups (Supplementary Tables S25-S28).

Supplementary Table S25. MRI – One-way ANOVA

MRI				
One-way Analysis of Variance				
Waveform	Effect	Degr. of (Freedom)	F	p
Axial Length	Intercept	1	35963.22	< 0.0001
	Genotype	3	2.28	0.0917
	Error	47		
Equatorial Diameter	Intercept	1	22894.15	< 0.0001
	Genotype	3	0.37	0.7777
	Error	47		
Lens Axial Length	Intercept	1	28798.93	< 0.0001
	Genotype	3	3.01	0.0395
	Error	47		
Lens Equatorial Diameter	Intercept	1	19942.68	< 0.0001
	Genotype	3	1.66	0.1873
	Error	47		
Global Asphericity	Intercept	1	31329.85	< 0.0001
	Genotype	3	1.01	0.3962
	Error	47		
Lens Asphericity	Intercept	1	59639.70	< 0.0001
	Genotype	3	1.27	0.2950
	Error	47		
Eye Distance	Intercept	1	16010.04	< 0.0001
	Genotype	3	3.66	0.0188
	Error	47		
Weight	Intercept	1	1864.024	< 0.0001
	Genotype	3	14.47	< 0.0001
	Error	47		

Supplementary Table S26. MRI: Lens Axial Length

MRI: Lens Axial Length				
Fisher's least significant difference (LSD) post hoc analysis				
Genotype	Genoytype			
	Control (Ts)	Ts656Dn	Control (Dp)	Dp16
Control (Ts)		0.6419	0.0140	0.9305
Ts656Dn	0.6419		0.0055	0.6000
Control (Dp)	0.0140	0.0055		0.0232
Dp16	0.9305	0.6000	0.0232	

Supplementary Table S27. Eye Distance

Eye Distance				
Fisher's least significant difference (LSD) post hoc analysis				
Genotype	Genoytype			
	Control (Ts)	Ts656Dn	Control (Dp)	Dp16
Control (Ts)		0.0426	0.1789	0.9937
Ts656Dn	0.0426		0.0021	0.0535
Control (Dp)	0.1789	0.0021		0.2033
Dp16	0.9937	0.0535	0.2033	

Supplementary Table S28. Mouse Weight

Mouse Weight				
Fisher's least significant difference (LSD) post hoc analysis				
Genotype	Genoytype			
	Control (Ts)	Ts656Dn	Control (Dp)	Dp16
Control (Ts)		< 0.0001	0.2427	0.4331
Ts656Dn	< 0.0001		< 0.0001	0.00011
Control (Dp)	0.2427	< 0.0001		0.0693
Dp16	0.4331	0.00011	0.0693	
